# Structure-based identification of novel inhibitors targeting the enoyl-ACP reductase enzyme of *Acinetobacter baumannii*

**DOI:** 10.1038/s41598-023-48696-z

**Published:** 2023-12-04

**Authors:** Shama Khan, Shabir A. Madhi, Courtney Olwagen

**Affiliations:** 1 South African Medical Research Council: Vaccines and Infectious Diseases Analytics Research Unit, Faculty of Health Science, School of Pathology, University of the Witwatersrand, Johannesburg, South Africa; 2 Wits Infectious Diseases and Oncology Research Institute, Faculty of Health Science, University of the Witwatersrand, Johannesburg, South Africa

**Keywords:** Computational biology and bioinformatics, Drug discovery

## Abstract

*Acinetobacter baumannii* is a Gram-negative multidrug-resistant bacterial pathogen primarily associated with nosocomial infections resulting in increased morbidity and mortality in adults and infants, especially in sub-Saharan Africa where the clinical burden is high. New therapeutics are needed to treat multidrug-resistant *Acinetobacter baumannii* infections and reduce transmission. The study used computer-integrated drug discovery approaches including pharmacophore modelling, molecular docking, and molecular dynamics simulation to screen potential inhibitors against the enoyl-acyl carrier protein reductase—FabI protein of *Acinetobacter baumannii*. The top three potential inhibitors: 21272541 > 89795992 > 89792657 showed favourable binding free energies including coulombic energy, van der Waals energy, and polar and non-polar energies. Furthermore, all three complexes were extremely stable and compact with reduced fluctuations during the simulations period. Inhibitor 21272541 exhibited the highest binding affinity against the *Acinetobacter baumannii* FabI protein. This is similar to our recent report, which also identified 21272541 as the lead inhibitor against *Klebsiella pneumoniae* infections. Future clinical studies evaluating drug effectiveness should prioritise inhibitor 21272541 which could be effective in treating infections caused by Gram-negative organisms.

## Introduction

*Acinetobacter baumannii* (*A. baumannii*) is a Gram-negative opportunistic organism associated with nosocomial bloodstream, urinary tract and airways infections, particularly in immunocompromised individuals and in intensive care units (ICU) facilities. Globally, *A. baumannii* is responsible for 1.27 million annual deaths, with the burden of disease being highest in Sub-Saharan Africa (27·3 deaths per 100 000)^1^ where there is limited access to healthcare resources. The World Health Organization (WHO) has identified *A. baumannii* as a multidrug-resistance (MDR) critical priority pathogen as it causes deadly bloodstream infections^2^, which in the absence of preventive measures such improved infection control and development of vaccines or new antimicrobial drugs, could potentially contribute to 10 million deaths annually by 2050.

*A. baumannii* forms a part of the ESAKPE (***E****nterococcus faecium*, ***S****taphylococcus aureus*, ***K****lebsiella pneumoniae*, ***A****cinetobacter baumannii*, ***P****seudomonas aeruginosa*, and ***E****nterobacter spp.*) organisms which are associated with infections acquired in hospitals (HAI); and are characterised by high levels of resistance towards multiple classes of antibiotics leading to reduced therapeutic options^3^. Antimicrobial resistance has spread globally through various mechanisms including extended-spectrum β-lactamases (ESBLs), carbapenemases and AmpC beta-lactamases enzymes (AmpC)^4^. The high prevalence of both extreme drug resistant (XDR) and pan drug resistant (PDR)^5^ *A. baumannii* strains poses a challenge for clinical therapeutics. Furthermore, MDR *A. baumannii* is often associated with inappropriate empiric therapies which contribute to high case fatality rates^6^. *Acinetobacter baumannii* isolates manifesting XDR are defined as being highly resistant to three or more classes of antibiotics, including fluoroquinolones, penicillins, cephalosporins with inhibitor combinations and aminoglycosides. Resistance of *A. baumannii* isolates to carbapenems (i.e., PDR) is associated to polymyxins and tigecycline antimicrobial class of drugs^7^. There is a paucity of data on invasive *A. baumannii* disease cases due to PDR strains, which is associated with severe illness, multi-morbidity, long hospital admissions and exposure to multi-invasive procedures^8^. Although some developments of new antibiotics targeting Gram-negative bacteria are currently underway, additional antimicrobial therapy options are required as spread of MDR, XDR and PDR *A. baumannii* strains continues to emerge as a major health concern^9^.

All Gram-negative bacteria have a distinctive outer membrane composed of glycolipid lipopolysaccharides and glycerol phospholipids that acts as a protective barrier against antibiotics or other toxic compounds^10^. Fatty acids can be formed through two different pathways containing type I and type Fatty Acid Synthesis (FASI and FASII). FASI is achieved by an enzyme using a large multidomain and multifunctional structure in mammals whereas FASII pathway utilized by bacteria involving chains of distinct and functionally specific enzymes producing fatty acids which ordained to integrate in membrane lipids. Acyl-carrier protein (ACP) is attached to the acyl chain through a thioester linkage and accountable to transport the substrate among enzymes of the FASII pathway. The pathway then initiates with the condensation of acetyl-CoA and malonyl-ACP via Fatty acid biosynthesis enzyme-FabH. This step is followed by eliminating β-keto group by FabG, dehydration of the substrate by FabZ or FabA, and then reduction by FabI, FabL, FabK, or FabV. Condensation by FabB or FabF commences a new cycle which repeats, elongating the fatty acid chain by two carbons each cycle. Hence, novel antimicrobial drugs should be designed targeting non-homologous bacterial enzymes due to differences in FASI and FASII pathways. Bacteria’s can shelter several FabI isoforms (viz. FabI/FabL/FabK/FabV), however *A. baumannii* only contains a single FabI enzyme^11^ involved in FAS-II^12^ present in the cytoplasmic membrane. Therefore, new therapeutics should be focused on inhibiting the biological targets located in the cytoplasmic membrane of Gram-negative organisms. The NADH dependent FabI catalyses the last reaction in FAS-II by converting trans-2-enoyl-ACP to acyl-ACP and removing the double bond at second position of a fatty acid chain^13^. The enzyme also involved in the fatty acid elongation cycle, which is essential in lipid metabolism and biotin biosynthesis^14^. The role of FabI in catalysing FAS-II in *A. baumannii* makes it a favourable target for the discovery and development of novel antimicrobial agents^15^. Triclosan is a widely used antimicrobial agent and blocks the activity of bacterial FAS-II^16^, hence, we have used *A. baumannii* FabI protein in complex with triclosan and NADH as a model system to design *A. baumannii* specific inhibitors. This study focuses the advancement of novel antimicrobial agents targeted at the *A. baumannii* FabI protein.

## Computational methodologies

### Protein structure preparation

The structure of the *A. baumannii* FabI protein in complex with triclosan and NADH was retrieved from the Protein Data Bank (PDB) database^17^ under the accession number of 6AH9 (https://www.rcsb.org/structure/6ah9) and used as a modelling system to perform molecular docking and extended molecular dynamics simulations studies. The structure of the protein was processed, minimized and taken for refinement using Protein Preparation Wizard^18^ in Maestro 13.3 version of Schrodinger package. The charge and protonation state were equivalent to pH 7.0 on the protein structure considering all basic and acidic amino acids. Afterwards, energy minimization was applied with the liquid simulations force field (OPLS-2005) on protein’s structure with root mean square deviation (RMSD) cut-off 0.30 Å to remove any steric clashes from the residues after adding hydrogen atoms and hetero-groups.

### Ligand structure preparation

Triclosan was used as a model compound to screen a library of available compounds from PubChem database^19^. PubChem is a public repository for chemical compounds with their biological activities as this information lacks in other databases like ZINC. This database is also integrated in other biomedical database hosted by National Institutes of Health (NIH) including PubMed, Protein, Gene, BioAssay etc. making it more relevant in comparison with other publicly available databases^20^. The ligand-dataset of 140 compounds were filtered based on a 90% similarity index including Lipinski’s rule of Five^21^ from the database. This virtual library was then taken for comprehensive screening evaluations. All chemical structures including triclosan were prepared using the LigPrep module^22^ of the Schrodinger package involving addition of hydrogen atoms, selecting correct orientation, ring conformations and ionization states. The liquid simulations force field (OPLS-2005) was used to apply partial charges on the compounds and the compounds were then exposed to energy minimization until RMSD reached to 0.001 Å. Epik ionization^23^ tool was used to neutralize the pH of all compounds.

### Identification of pharmacophore hypotheses

The structure-based e-pharmacophore strategic approach was considered using the PHASE^24^ modules of Schrodinger package. Hydrogen bond acceptor (A), hydrogen bond donor (D), hydrophobic contacts (H), negative ionizable (N), positive ionizable (P), and aromatic ring (R) were selected as default chemical features to generate the most illustrative features of the active site of *A. baumannii* FabI protein. Hypothesis Generation for Energy-Optimized Structure Based Pharmacophores (e-Pharmacophore) was used to process the features with excluded 5 Å volume of the refined inhibitor for the FabI protein. Pharmacophore features were then selected based on the critical interactions with the key amino acids of protein involved around the pharmacophore inhibitor. The generated pharmacophore characteristics contained all functional groups to inhibit the activity *A. baumannii* FabI protein.

### Screening of the A. baumannii FabI inhibitor-like compounds using docking-based virtual screening

To screen the library of 140 compounds extracted from the PubChem database against *A. baumannii* FabI protein, the obtained 3D pharmacophore features were exported and established as a reference for the PHASE-based virtual screening protocol to identify potential compound against FabI protein. A total of 136 (97%) out of 140 compounds were selected for further analyses based on their highest negative PHASE scores and the identical attributes of the compounds were also considered using the Phase Screen Scores. The Glide-based virtual screening module^25^ of Schrodinger package was used to filter the lead compounds that strongly interacted by producing multiple number of non-covalent interactions with the *A. baumannii* FabI protein. The grid around the active site of receptor was built with a big cubical box (28 × 28 × 28 Å) and a small cubical box (20 × 20 × 20 Å) for accurate binding. The library was then docked in a three-step docking protocol with High throughput Virtual Screening (HTVS) followed by Standard Precision (SP) and then Extra-Precision (XP) modes^26,27^. The Docking-Based Virtual Screening (DBVS) protocol was used to filter all 136 compounds. Twenty-three (32%) were chosen for further analyses based on the XP-GScore. The docked complexes were ranked based on their binding scores and best interaction poses. The stability of these complexes assumed to have higher potential as their binding energies were having negative scores.

### Ligand-based ADME/toxicity properties assessment

The Absorption, Distribution, Metabolism, Excretion and or Toxicity (ADME/T) analysis on the 23 filtered compounds was performed using QikProp 5.6 module^28^ in the Schrodinger package to generate the related descriptors. Briefly, QikProp assess significant descriptors based on pharmacokinetic and physiochemical properties of the compounds. The ADME/T analysis includes membrane permeability (log P), blood–brain barrier (log BB), central nervous system (CNS), solvent surface accessible area (SASA), solubility (log S) and percentage of human oral absorption of the compounds. All these factors are important to filter a potential compound to be an ideal drug. The acceptable range for CNS is from–2 (inactive) to + 2 (active), for SASA it should be in a range from 300 to1000, for octanol/water partition coefficient the standard acceptable range is between 2 to 6.5, for aqueous solubility the normal range is − 6.5 to 0.5 mol/dm, for BB the drug candidate should be fall between − 3.0 and 1.2 and the percentage of human oral absorption must be < 25% and > 80%. The processed ADME/T properties^27^ define the drug-likeness activity of the filtered compounds leads to the selection of top three hit compounds: 21272541, 89795992, 89792657 which further directed towards the Molecular dynamic (MD) simulations for extended analysis.

### Molecular dynamic (MD) simulation studies

The elaborated method for MD simulation has already been described in our recently published report^29^. Briefly, the Assisted Model Building with Energy Refinement (AMBER) 18 tool^30^ was used to perform 200 ns of MD simulations on the four complexes: 21272541-FabI, 89795992-FabI, 89792657-FabI with triclosan-FabI as a model system. Graphics Processing Unit (GPU) accelerated version of Partial Mesh Ewald Molecular Dynamics (PMEMD) was used for fast and accurate simulations^31^. The general AMBER force field (GAFF)^32^ ff14SB was used for ligand–protein parametrization and inbuilt parameters for NADH co-factor was used with the LEaP module^32^ of AMBER 18. Antechamber module with GAFF force field was used to assign the partial charges and to generate the atom types for each molecule. Furthermore, the LEaP module was used to generate the TIP3P water box of 10 Å, neutralize the systems with sodium and chloride ions with neutral charge, and to create the topology and input coordinate files for simulation studies. All four systems were then taken for 200 ns MD simulations to analyse the behaviour of our chosen compounds against *A. baumannii* FabI drug discovery.

### Post-dynamic trajectory analysis

After the completion of the MD simulation, trajectories were analysed with CPPTRAJ module^33^ implemented in AMBER 18 tools. It is the main program in AMBER for processing coordinate trajectories to perform complicated analysis. The root mean square deviation (RMSD) of Cα atoms, root mean square fluctuation (RMSF) of amino acid residues in the complex, radius of gyration (RoG), solvent accessible surface area (SASA), intra-molecular hydrogen bond, distance correlation matrix and principal component analysis was performed with this module. For scheming the 2-dimensional (2D) plots of the trajectories, Origin 7.0 graphic and data analysing tool^34^ was used.

### Binding free energy calculation and per-residue free energy decomposition

Molecular Mechanics/Generalized Born Surface Area (MM/GBSA)^35^ is a widely used technique to calculate free energies of the bound ligand to its associated protein/s. CPPTRAJ module was used to strip all water molecules, ions and solvents from the trajectories. The free energies (ΔG_bind_) were computed using below equation for all four systems:

1 $$\Delta G_{bind} = \, G_{complex} {-}G_{protein} {-}G_{ligand}$$

The free energy term, Δ*G*_*bind*_ is computed using the following equations:

2 $$\Delta G_{bind} = \, \Delta E_{gas} + \, \Delta G_{solvation} {-}T\Delta S$$

where

3 $$\Delta E_{gas} = \, \Delta E_{int} + \, \Delta E_{vdw} + \, \Delta E_{elec}$$

4 $$\Delta E_{int} = \, \Delta E_{bond} + \, \Delta E_{angle} + \, \Delta E_{torsion}$$

5 $$\Delta G_{solvation} = G_{polar} + G_{nonploar}$$

6 $$\Delta G_{nonploar} = \gamma \Delta SASA + \beta$$

Per-residue free energy decomposition was used to decompose all the energies of key contributing amino acid residues for each ligand. The decomposition energy describes the contribution of binding residues of protein with its ligand.

### Distance correlation matrix analysis

To validate the residual movements of the *A. baumannii* FabI protein in complex with all ligands, the dynamic cross correlation matrix (DCCM) method was used which describes both the positive and negative movements of amino acid residues in the protein–ligand complexes and to provides information on the contribution of Cα backbone atoms of protein after ligand binding as described previously^36^.

### Principal component analysis

Principal component analysis (PCA) was chosen to categorise the conformational modifications in protein’s structure that aid in explaining the motions of all residues after protein–ligand binding^37^. Correlated and anti-correlated fluctuations can be observed in the trajectory through this analysis. These fluctuations in the amino acids were probed with covariance matrix C defined on the atomic coordinates and eigenvectors. The eigenvectors and eigenvalues are stated as the directions and the degree of residual motions correspondingly^29^. An overall RMSD was calculated after aligning the selected compounds with a reference model system using the least-square fit process to remove all translational and rotational motions in the trajectories. The 3-dimensional (3D) porcupine structure of the protein’s collective motions was drawn with Normal Wizard mode analysis in VMD tool^38^.

## Result and discussion

### Database screening

The chemical database was screened based on a 90% similarity index to triclosan and Lipinski’s Rule of five^21^ revolution to receive a library of 140 compounds. The library is presented in Table S1 with their associated smile structures and PubChem identifiers. This library was chosen as the primary input for extended studies in this report.

### Structure‑based pharmacophore modelling

**Figure 1:**
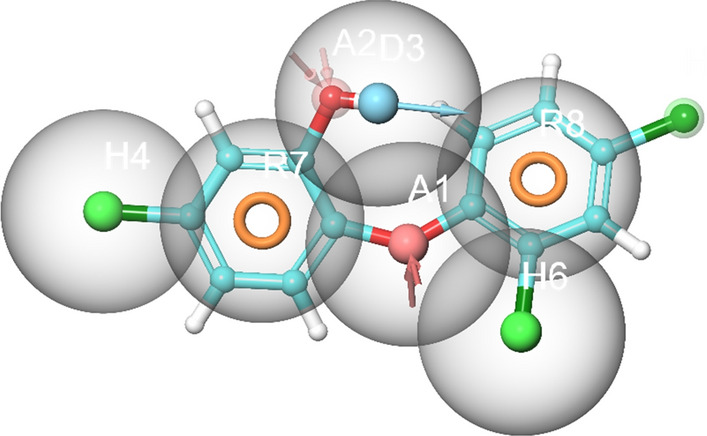
Structure-based 3D pharmacophore modelling based on the analysis of triclosan interactions with the *A. baumannii* FabI protein and its chemical structure surrounded by excluded volumes. Red arrow: Hydrogen bond acceptor, blue arrow: Hydrogen bond donor, orange sphere: aromatic ring.

Structure-based e-pharmacophore modelling of the 3D target protein is useful in providing accurate knowledge of protein–ligand interactions^39^. Default descriptors were used to generate the best e-pharmacophore model. The key contributing residues involved in ligand binding were imported in generating e-pharmacophore of triclosan-FabI complex. The subsequent e-pharmacophore model for *A. baumannii* FabI protein is characterized with multiple features including H-bond donor, H-bond acceptor, and π–π stacking of aromatic rings as shown in Fig. 1.

### Virtual screening of the selected compounds

The attained characteristics of the e-pharmacophore triclosan model in complex with *A. baumannii* FabI protein were selected as input to filter the prepared library of 140 compounds and are recorded in Table S1. A total of 136 compounds (Table S2) passed the filtering parameters based on the designed e-pharmacophore hypothesis.

### Docking‑based virtual screening analysis

The screened 136 compounds were used as input for DBVS as detailed in the methodology. The Glide SP protocol successfully identified 130 compounds (Table S3) following by Glide XP protocol which further narrowed the numbers of compounds to 71 (Table S4) with increased binding scores. These 70 compounds were then optimized to produce one pose per ligand based on the most negative binding score (− 8.03 kcal/mol) against the *A. baumannii* FabI protein. The top three lead candidate compounds i.e., 21272541, 89795992 and 89792657 underwent ADME/T analysis and these compounds were taken for extended MD simulation studies in process to develop novel inhibitors for *A. baumannii* infections.

### Molecular docking analysis

**Figure 2:**
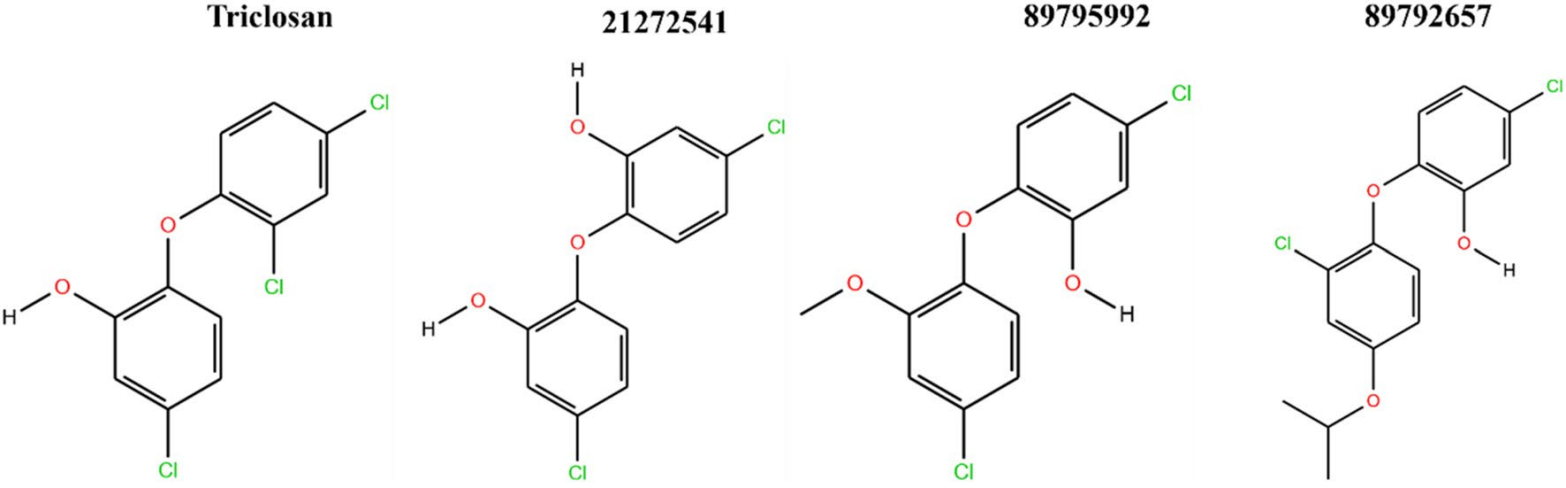
2D structures of model inhibitor triclosan with top three lead compounds.

The top three hit leads (21272541, 89795992, 89792657) with triclosan in complex with the *A. baumannii* FabI protein were selected for molecular docking studies to check their initial stability in the form of interactions and binding scores. The four 2D structures of model compound triclosan with their 90% similar derivative are presented in Fig. 2.

**Figure 3:**
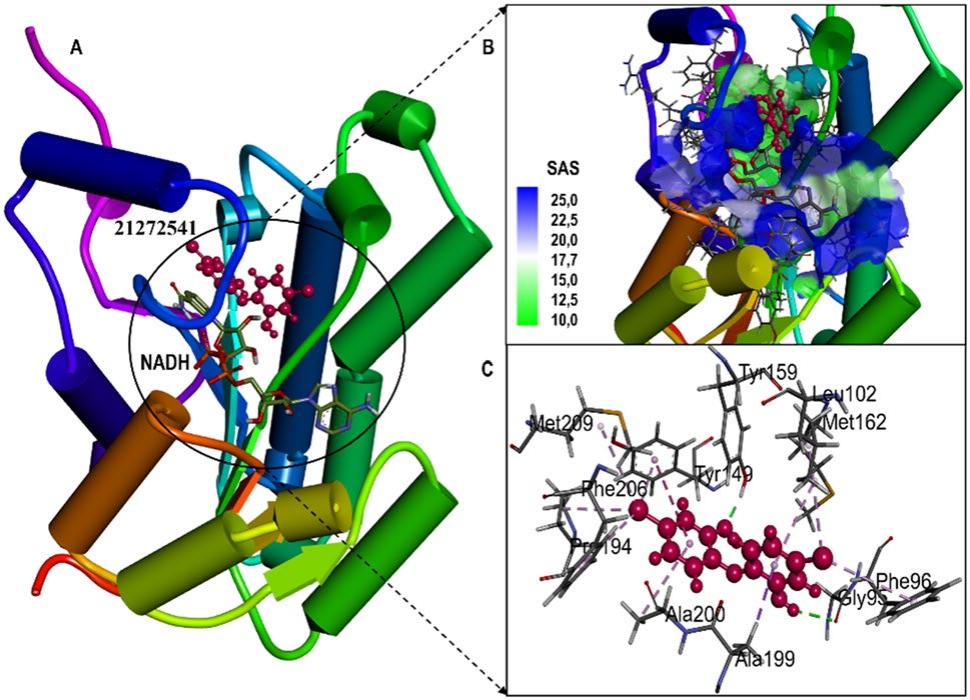
(**A**) Structural representation of *A. baumannii* FabI protein in complex with newly developed inhibitor 21272541; (**B**) Zoomed in view of the binding site residues coloured by solvent accessible surface area of FabI accommodating 21272541 and NADH, (**C**) Close up view of active residues interacting with the inhibitor showing hydrogen bonds and other Pi-Pi interactions.

**Figure 4:**
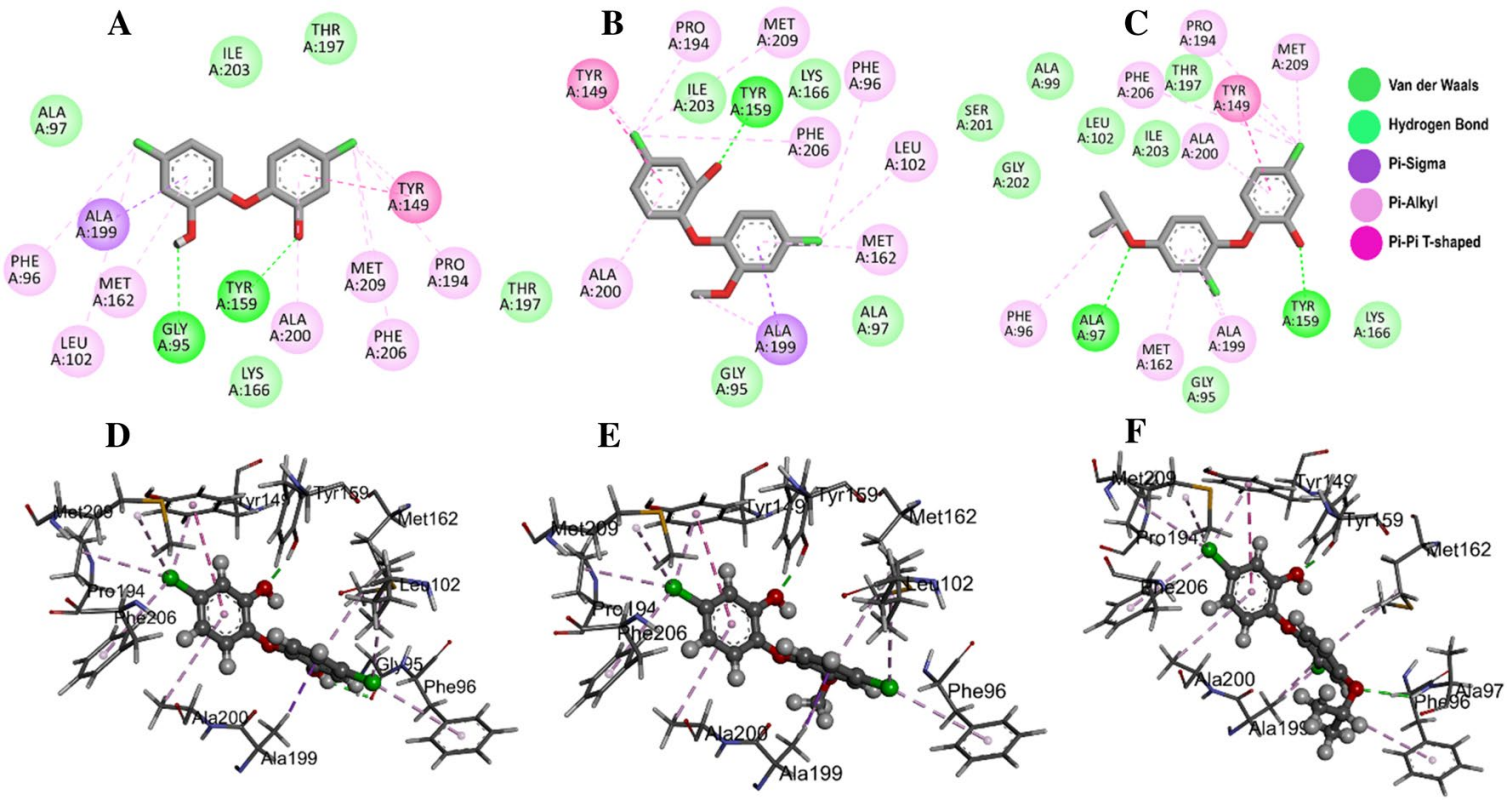
Molecular docking of the FabI protein with the proposed inhibitors: 21272541, 89795992 and 89792657. (**A–C)** Molecular interactions of the *A. baumannii* FabI protein with studied compounds with their 2D maps after docking and **(D–F)** their associated 3D interactions.

The active site amino acid residues involved in the binding of selected compounds with *A. baumannii* FabI protein are shown in Table 1 with their associated binding scores. The binding score for compounds 21272541, 89795992, and 89792657 are − 9.84, − 9.65 and − 9.56 kcal/mol, respectively and were indicative of strong binding with the FabI protein. Furthermore, these scores were higher than the model compound which revealed the binding score of − 8.34 kcal/mol. The binding mode of 21272541 with other two compounds in 3D images and 2D plots are illustrated in Figs. 3 and 4.

**Table 1:** Docking score and residues involved in binding of studied compounds and model drug in complex with *A. baumannii* FabI protein. Bold highlighted residues are immersed in creating major interaction networks.

Protein–ligand Complexes	Docking score (Kcal/mol)	Interacting residues
Triclosan-FabI	− 8.34	GLY95, PHE96, ALA97, LEU102, TYR149, **TYR159**, MET162, LYS166, PRO194, THR197, ALA199, ALA200, ILE203, PHE206, MET209
21272541-FabI	− 9.84	**GLY95**, PHE96, ALA97, LEU102, TYR149, **TYR159**, MET162, LYS166, PRO194, THR197, ALA199, ALA200, ILE203, PHE206, MET209
89795992-FabI	− 9.65	GLY95, PHE96, ALA97, LEU102, TYR149, **TYR159**, MET162, LYS166, PRO194, ALA199, ALA200, ILE203, PHE206, MET209
89792657-FabI	− 9.56	GLY95, PHE96, **ALA97**, ALA99, LEU102, TYR149, **TYR159**, MET162, LYS166, PRO194, THR197, ALA199, ALA200, SER201, GLY202, ILE203, PHE206, MET209

Multiple interactions were identified in all the studied compounds (Fig. 4A–C**).** Compound 21272541 might be the lead inhibitor as it produced two hydrogen bonds with Gly95 and Tyr159. The strong interaction network of the Pi-Sigma was also observed with compound 21272541 and Tyr149 residues which was a key contributing residue in the *Klebsiella pneumoniae* 21272541-FabI complex in our previous study^29^. This compound also contributed to the binding by producing Pi-Alkyl interaction which helps in the stability of the complex. The second lead compound 89795992 only formed one hydrogen bond with residue Tyr159 with various Pi-Sigma and Pi-Alkyl networks. While compound 89792657 formed two hydrogen bonds with Ala97 and Tyr159 (Fig. 4D–F). Furthermore, Ala199 was involved in the formation of Pi-Sigma interaction with the benzene ring in all three compounds. The referent inhibitor triclosan only formed one hydrogen bond with Tyr159 and one Pi-Pi stacking with Tyr149 (Figure S1). Tyr159 was thus identified as the key contributor to the binding of *A. baumannii* FabI protein as it was involved in the hydrogen bond formation of all the three compounds. Nevertheless, compound 21272541 showed the most favourable binding score and was majorly involved in multiple interaction network within the *A. baumannii* FabI protein and could thus be considered as potential drug candidate after performing extended *in-silico* studies.

### ADME/T analysis

The evaluation of “drug-likeness” properties of the selected compounds with referent inhibitor triclosan were analysed to check if these compounds passed the standard filter criteria. The physicochemical and toxicity characteristics including cell permeation, the influence of metabolism, bioavailability, and toxicity, of the three compounds together with triclosan is detailed in Table 2. We have checked the mentioned characteristics for triclosan with all three drug candidate compounds. Briefly, the projected central nervous system activity (CNS) activities were 1, 0, 1, 1 for triclosan, 21272541, 89795992, and 89792657, respectively and were all in the expected range as mentioned in ADME/T methodology.

**Table 2:** *In-silico* ADME/T predictions of the selected compounds.

Compounds	^a^CNS	^b^QPlogKhsa	^c^SASA	^d^QPlogPo/w	^e^QPlogS	^f^QPlogBB	^g^% Human oral absorption
Triclosan	1	− 0.91	356.54	3.67	− 5.20	− 0.87	59.94
21272541	0	0.06	472.66	6.16	− 3.89	− 0.20	100.00
89795992	1	0.31	494.18	5.31	− 4.50	0.25	100.00
89792657	1	0.53	539.31	5.04	− 5.05	0.17	100.00

^a^Predicted central nervous system activity from –2 (inactive) to + 2 (active).

^b^Prediction of binding to human serum albumin (acceptable range: − 1.5–1.5).

^c^Total Solvent Accessible Surface Area: SASA (acceptable range: 300–1000).

^d^Predicted octanol/water partition coefficient (acceptable range: 2–6.5).

^e^Predicted aqueous solubility, S in mol/dm (acceptable range: − 6.5–0.5).

^f^Predicted brain/blood partition coefficient (acceptable range: − 3.0–1.2).

^g^Predicted percentage human oral absorption (< 25% is poor and > 80% is high).

Furthermore, the estimated digital values of the human binding serum albumin (QPlogKhsa) of all compounds are in the acceptable range except for triclosan which was − 0.91. The solvent accessible surface area (SASA) of all candidate inhibitors and triclosan was in the acceptable range of 300–1000; however, triclosan had the lowest SASA (356.54) compared to compound 89792657 which exhibited the highest SASA (539.31). Compound 21272541 was identified to have the most suitable octanol/water partition coefficient (QPlogPo/w; (6.16) compared with the other inhibitors which had lower values, albeit still in the expected range of between 2 and 6.5. The aqueous solubility (QPlogS) estimates and the brain/blood partition coefficient (QPlogBB) for all inhibitors with the referent inhibitor were in the acceptable range of − 6.5 to 0.5 and − 3.0 to1.2, respectively. Lastly, all three inhibitors showed 100% human oral absorption (%) except for triclosan which only showed 59.94% human oral absorption and was below the acceptable range (< 25% and > 80%). Nevertheless, candidate compounds pointed towards favorable drug-likeness properties compared to the model inhibitor and could be promising inhibitors of the *A. baumannii* FabI protein.

### Post‑dynamics MD trajectories analysis

The 3D structure of the protein endures major changes after binding of compatible inhibitor which have great effect on proteins biological activity^40^. In disease effected pathways, the protein-inhibitor binding influences the mode of inhibition of the target protein, thus, analysing the dynamics, fluctuations, and compactness of the protein structure after binding the studied inhibitors was crucial to draw conclusions. After performing 200 ns of MD simulations with selected compounds and triclosan in complex with *A. baumannii* FabI complex, we analysed the trajectories to observe structural changes. The RMSD of Cα atoms was estimated for 200 ns for all four systems to observe the structural variations in the form of stability and competency.

**Figure 5:**
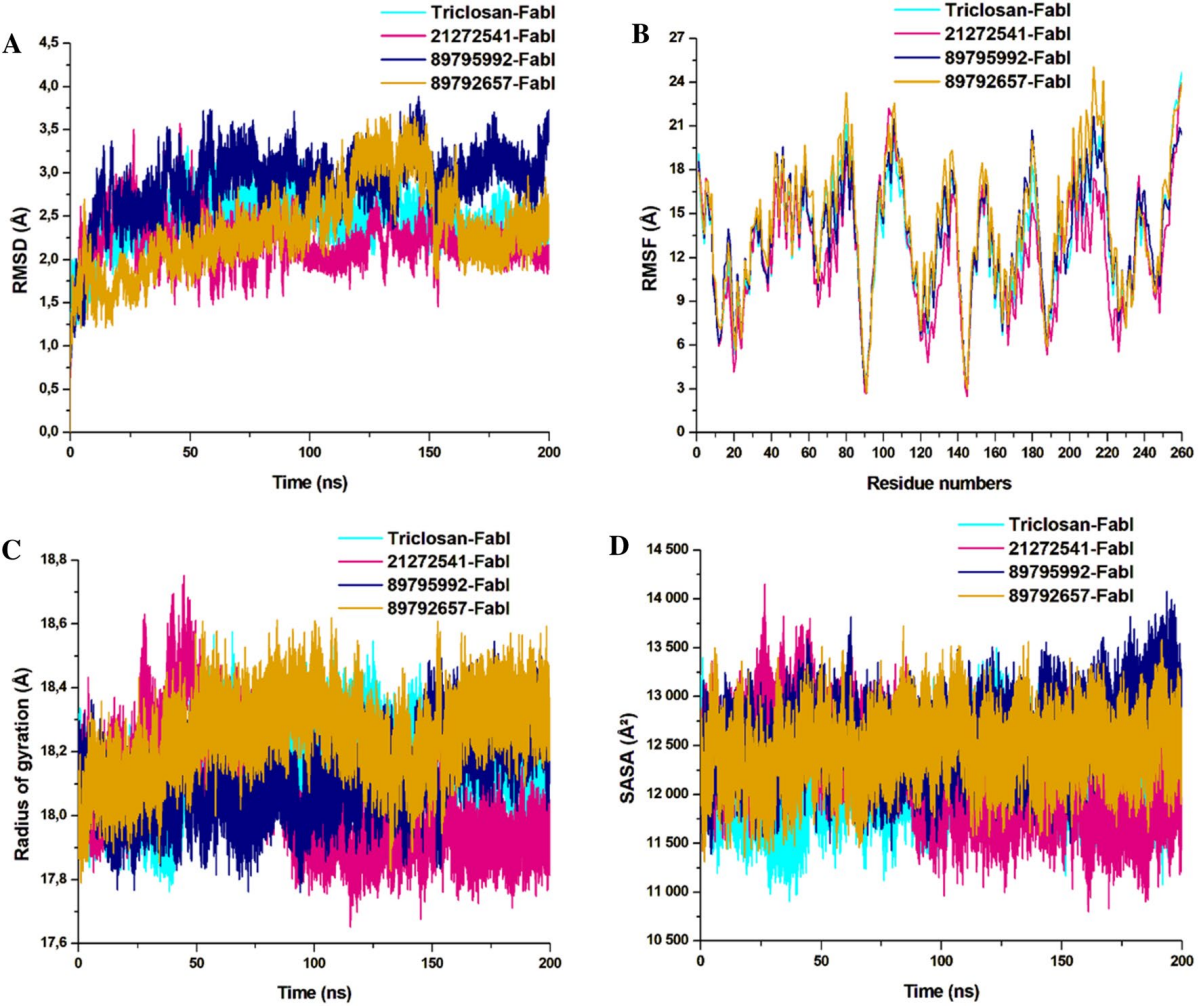
Structural dynamics of *A. baumannii* FabI protein–ligand complexes. (**A**) Cα backbone RMSD in Å of all the selected compounds bound to FabI protein; (**B**) RMSF values after compound binding; (**C**) RoG value after compound binding; (**D**) SASA values after compound binding after 200 ns of MD simulations.

The obtained RMSD values are plotted in the 2D lot and are sketched in Fig. 5A**.** All the systems were highly stable till the end of simulation and reached convergence after 50 ns except for the 89792657-FabI system which was unstable and only reached convergence after 150 ns. The 21272541-FabI complex was most stable revealing the RMSD value of 2.23 Å, followed by triclosan, 89792657 and 89795992 complexes with RMSD values of 2.51 Å, 2.89 Å, and 2.91 Å, respectively. Individually, all the systems were measured between 1 and 3.5 Å on the Y axis indicative of stable protein–ligand complexes. This analysis is an evidence that the systems were stable and further investigations performed on these trajectories would be reliable.

Furthermore, the RMSF of Cα atoms were calculated to observe the fluctuations in the amino acids in all four systems. Rigidity and flexibility were measured through this analysis. Complex 21272541-FabI was notably the least fluctuating in comparison with the other two complexes. The 89792657-FabI complex displayed high fluctuation values with 20.01 Å and was higher than the model system as represented in Fig. 5B**.** This analysis indicates that binding of 21272541 ligand induced least fluctuations in comparison with all other systems and this ligand could be feasible in *A. baumannii* FabI inhibition.

The RoG analysis was used to validate the folding and unfolding behaviour of the ligand–protein complexes and is crucial to determine the effect of micro molecule entities on the 3D target protein after their binding. The 21272541-FabI complex revealed the least RoG value of 18.02 Å and was stable after 50 ns of simulation time. The 2D plot of RoG can be seen from Fig. 5C. The 89792657-FabI complex revealed the highest RoG value of 18.34 Å indicative of less compactness and folding activities. All four systems; however, were between 17.81 and 18.5 Å values. Triclosan and 89795992-FabI complexes followed similar patterns of compactness. Better compactness and improved binding of selected compounds, specifically to 21272541 was offered with this analysis against *A. baumannii* FabI protein.

The last structural analysis included SASA which was performed to determine the hydrophobic and hydrophilic effects of the amino acids exposed to the solvent molecules during the MD simulations (Fig. 5D). The SASA values for all the systems were from 11,110 to 13,701 Å^2^. The 21272541-FabI complex showed the lowest SASA value and was also less exposed to solvents in comparison to other complexes. Furthermore, the triclosan-FabI complex was also similar to the 21272541-FabI complex; however, was slightly more exposed to the solvent compared with 89792657-FabI complex. Nevertheless, the alterations in the SASA values among the three selected compounds with model inhibitor are correlated with the folding and unfolding of the *A. baumannii* protein. Binding of the 21272541 compound is an indication of improved exposure to solvents and enhanced inhibition activity towards the FabI protein. The retention of all three ligands within the binding site of FabI protein was confirmed by generating snapshots initially at 10 ns and afterwards at every 50 ns till 200 ns of MD simulation as shown in Figure S2.

### Intramolecular hydrogen bond analysis

**Figure 6:**
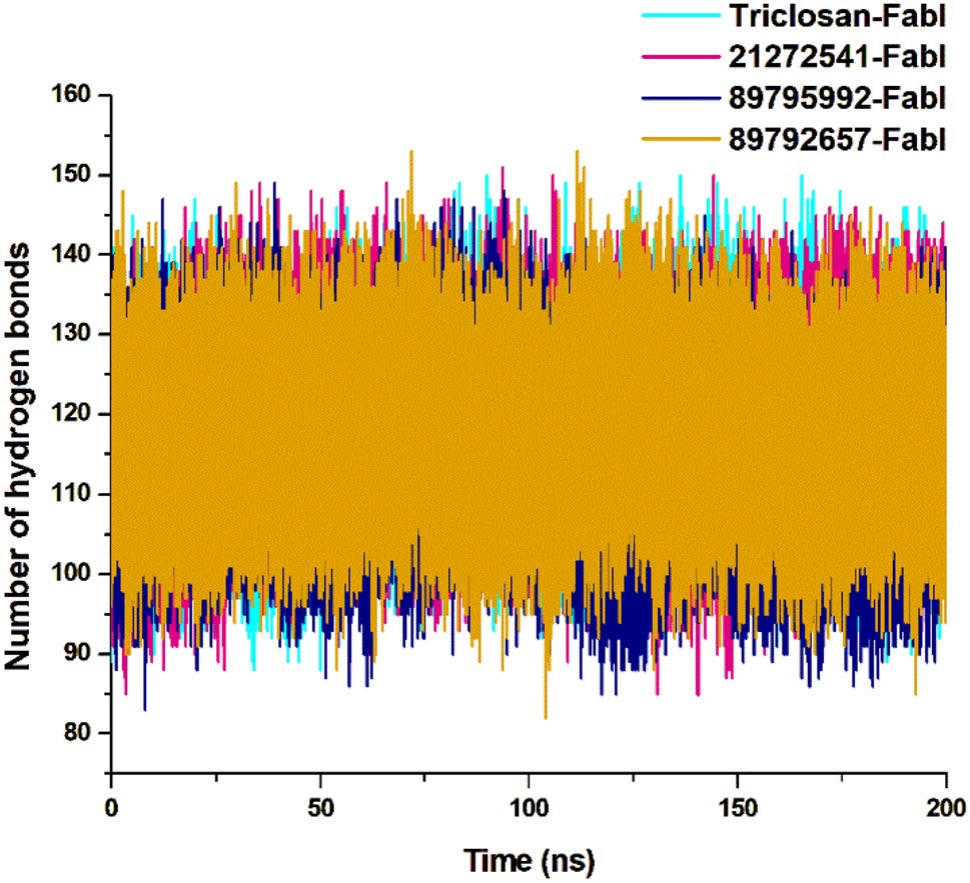
Intramolecular hydrogen bond analysis for studied complexes, calculated after 200 ns MD simulation.

The intramolecular hydrogen bond analysis is essential in estimating the conformation and stability of the protein. This evaluation extensively infers the mechanism of ligand–protein binding with better understanding in terms of hydrogen bond formation. The overall number of intramolecular hydrogen bonds in the 21272541-FabI complex were noted from 86 to 149 whereas in 89795992-FabI complex, it showed between 87 and 145. Minor differences were observed in these two complexes (Fig. 6), including a lower intramolecular hydrogen bonds of compound 89795992 compared to the triclosan-FabI complex. This intramolecular hydrogen bond analysis supports the above structural findings observed on *A. baumannii* FabI protein in complex with top three hits.

### Distance correlation matrix analysis

**Figure 7:**
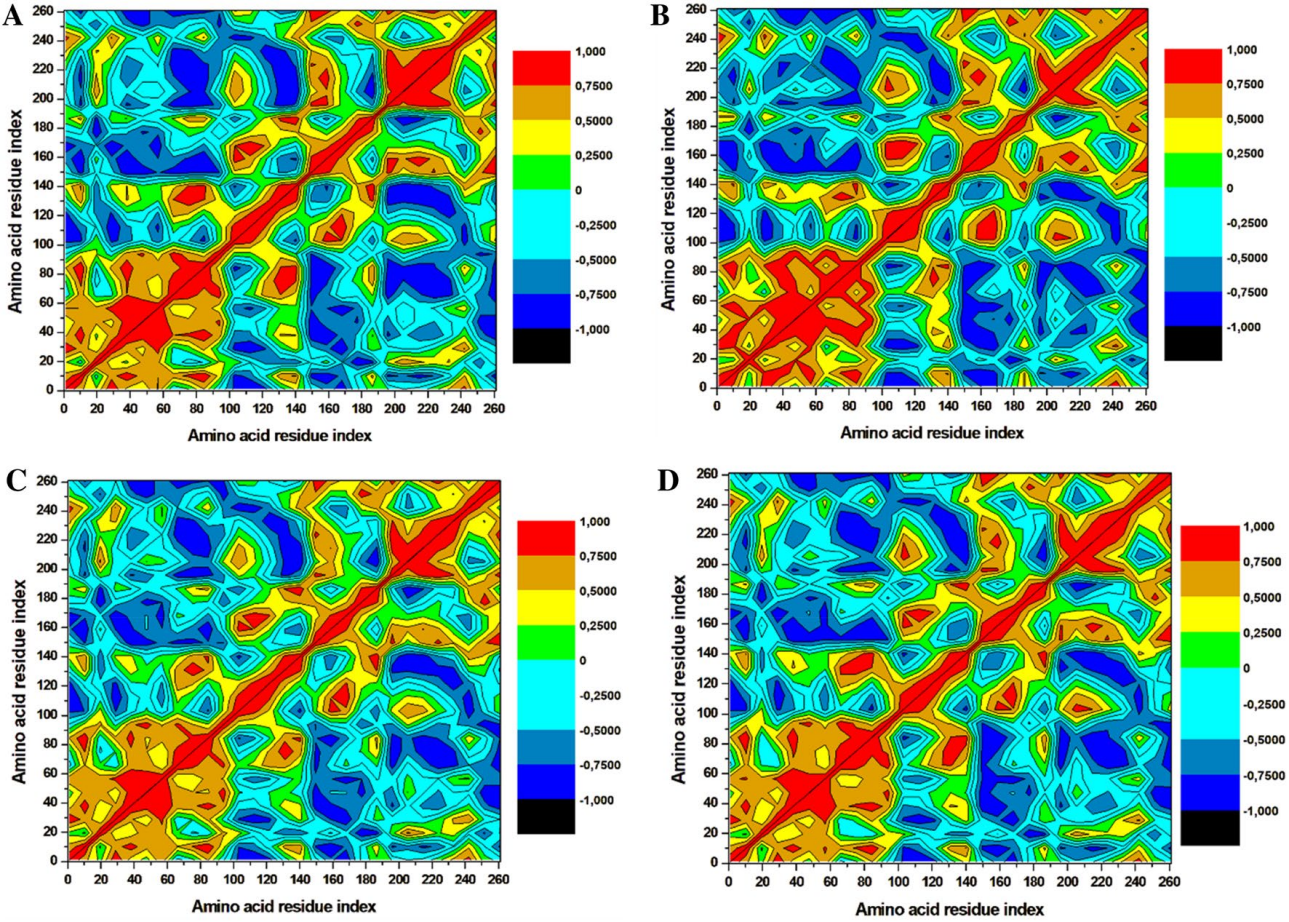
Dynamics cross-correlation matrix analysis. (**A**) Triclosan-FabI and (**B**) 21272541-FabI (**C**) 89795992-FabI and (**D**) 89792657-FabI complex calculated after 200 ns of MD trajectories.

The dynamics of the three selected compounds with triclosan in complex with the FabI protein were calculated by performing dynamic cross correlation matrix (DCCM) for the negative and positive residual correlation movements as shown in Fig. 7. The *A. baumannii* FabI protein can be seen dispersed into various positive and negative communities throughout the simulation. Triclosan-FabI, 89795992-FabI, and 89792657-FabI complexes followed a similar pattern of scattering; however, significant changes were noted in 21272541-FabI complex with mostly positive residual movement between the amino acids. Furthermore, the highly anti-correlated regions of the compounds range from 200 to 353 amino acids as they are not directly involved in the binding of selected ligands which could indicate lower inhibitory activity. Thus, this analysis suggests that the amino acids within the FabI protein are in positive correlation to 21272541 and this compound will most likely reduce the inhibition of the FabI protein.

### Principal component analysis

**Figure 8:**
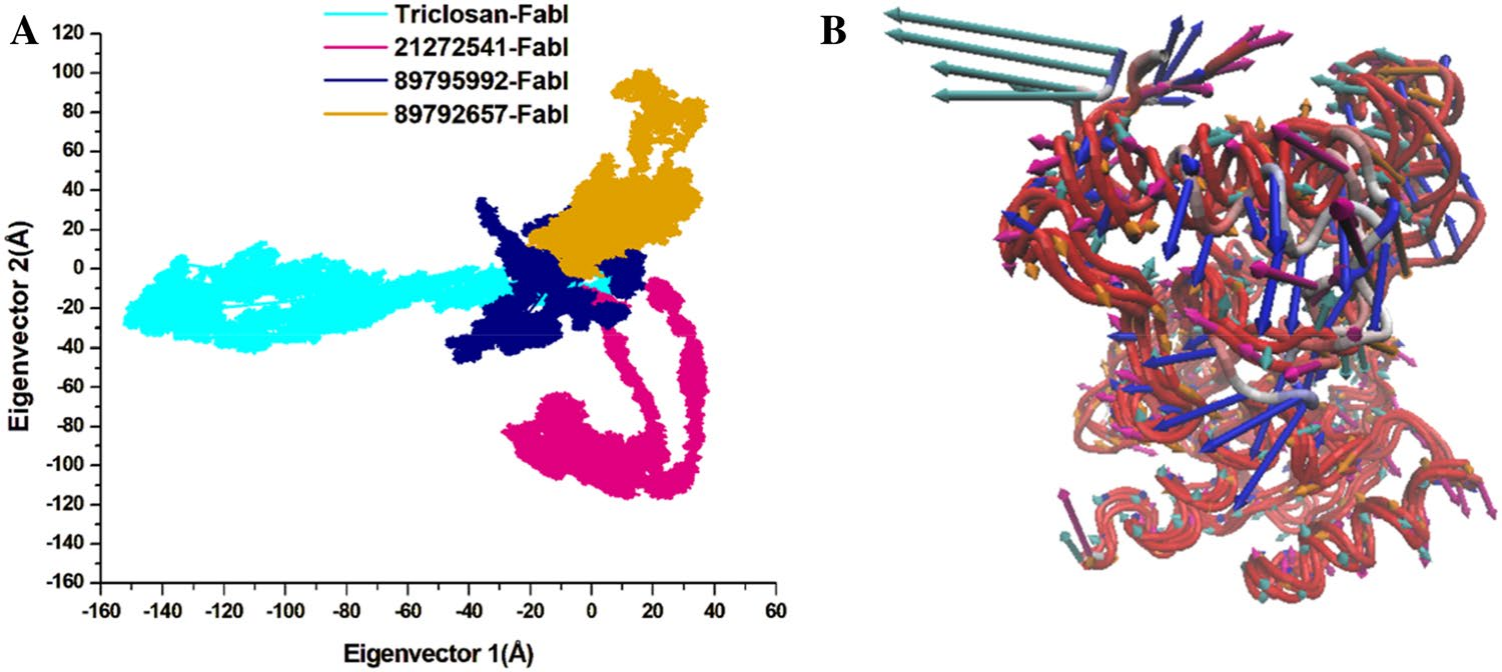
(**A**) PCA plot constructed by eigenvector 1 versus eigenvector 2 for the studied compounds with triclosan; (**B**) Porcupine plot of PC1 collective motions for the obtained predominant eigenvectors over the 200 ns MD trajectories for triclosan (cyan colour), 21272541 (pink colour), 89795992 and (blue colour), 89792657 (orange colour) and in complex with FabI protein.

The principal component analysis (PCA) was performed for the assessment of conformational modifications in amino acids upon ligand binding based on eigenvectors on the X and Y axis^29^. The continuous scattering of ligands in the PC1–PC2 points can be noted in the 2D plot in Fig. 8. Triclosan scattered in negative positions on PC1 whereas all three selected compounds are scattered on positive positions on PC1–PC2 eigenvectors. The 3D porcupine plot for all four systems was produced to interpret the ligands movements in various directions as displayed in Fig. 8. The compound 21272541 exhibited the most positive movements on eigenvector 1 on X axis; however, on the Y axis it showed a negative movement with a negative trace covariance matrix. It also displayed the trace covariance matrix of 20.18 Å with positive values on X axis. Compounds 89795992 and 89792657 denoted the positive correlation with least fluctuation on eigenvector 1 and 2 with positive values after binding to *A. baumannii* FabI protein. This analysis supports the previously mentioned DCCM analysis for positive and negative residual movements of complexes throughout the MD simulation.

### Binding affinity calculation with MM/GBSA

The overall binding affinities of various components of energies including electrostatic, van der Waal, polar, non-polar, solvation, and gas-phase energy were calculated with MM/GBSA technique. It is well-validated method to estimate the free energies involved in the binding of chemical molecules to biological macromolecules based on the MD simulation studies. The outcome from this analysis is recorded in Table 3. The 21272541-FabI complex revealed the most favourable free binding energy of − 61.63 kcal/mol as expected and was the most accurate compound with all the structural, hydrogen bond and PCA analysis. The model triclosan-FabI complex also showed better binding of − 59.02 kcal/mol in comparison with the 89795992-FabI and the 89792657-FabI complexes which exhibited the binding energies of − 52.09 kcal/mol and − 38.29 kcal/mol, respectively. The van der Waals energies was higher in the 89795992-FabI complex whereas it was lower in the 89792657-FabI with the digital values of − 46.68 kcal/mol and − 34.16 kcal/mol, respectively. Furthermore, the electrostatic energy was the highest in the 21272541-FabI complex whereas it was the lowest in 89795992-FabI complex with major difference of − 17.30 kcal/mol detected between the two compounds. The overall gas phase energies were favourable for all the studied complexes. The electrostatic and van der Waals energies are the most important contributors in ligand protein binding and they all showed promising results. The total binding energy is the difference of polar and non-polar energies and was accurately calculated as seen in the tabular results. The findings from all components of energies are indicative of significant binding of compound 21272541 to FabI protein and further validates this ligand to have the capacity to inhibit the activity of *A. baumannii* FabI protein and could potentially overcome drug resistant mechanisms.

**Table 3:** MM/GBSA-based binding energy profile of FabI in complex with top three studied compounds: 21272541, 89795992, 89792657 and triclosan.

Protein–ligand Complexes	∆E_vdW_	∆E_ele_	∆G_gas_	∆G_polar_	∆G_nonpolar_	∆G_sol_	∆G_bind_
Triclosan-FabI	− 45.02	− 59.23	− 92.03	65.37	− 6.21	59.16	− 59.02
21272541-FabI	− 45.47	− 60.45	− 95.87	66.25	− 3.81	65.44	− 61.63
89795992− FabI	− 46.68	− 43.15	− 87.98	60.64	− 4.39	56.25	− 52.09
89792657-FabI	− 34.16	− 51.21	− 88.01	55.01	− 8.17	46.84	− 38.29

### Per-residue energy decomposition analysis with MM/GBSA

The per-residue energy decomposition approach is commonly used to calculate the energy contribution of each amino acid and their association with the target protein in assisting rational of drug design. A cluster of 15 common amino acids were selected based on their involvement in the binding of 21272541, 89795992 and 89792657 compounds and model drug triclosan with *A. baumannii* FabI protein.

**Figure 9:**
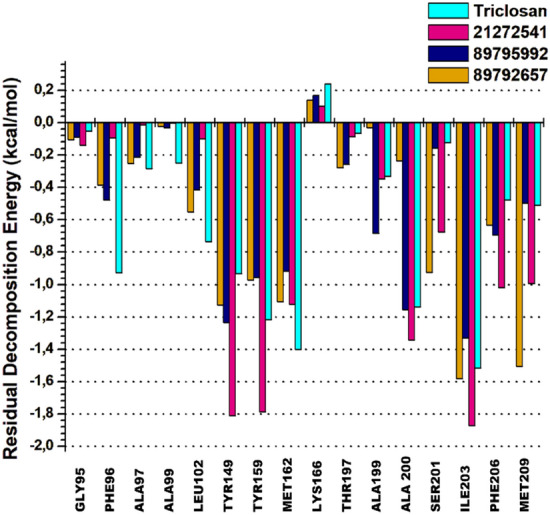
2D plot of per-residual energy decomposition of *A. baumannii* FabI protein in complex with the studied compounds and model drug triclosan.

Tyr149 and Tyr159 are key residues in the 21272541 binding with the most promising energies of − 1.81 kcal/mol and − 1.73 kcal/mol, respectively. Furthermore, these residues were involved in the formation of hydrogen bonds and Pi-Alkyl interactions with each studied ligands (Fig. 9**)**. Residue Ile203 also showed better binding energy of 21272541 with value of − 1.84 kcal/mol compared to referent inhibitor with value of − 1.54 kcal/mol; however, this residue did not produce any interaction network. The least contributing amino acids were from model drug triclosan exhibiting lower residual energies as compared to our screened compounds. Thus, from this analysis we observed the contribution of specific residues involved in strong binding.

## Conclusion

*A. baumannii* is a MDR bacterial pathogen and a leading cause of HAI in adults and infant with a high mortality ratio and limited therapeutic options to treat invasive *A. baumannii* infections. Alternative therapeutics are needed to treat MDR and XDR *A. baumannii* infections, especially in low-middle income countries where the burden of disease is the highest. This report is an effort to study the FabI protein in the context of drug discovery against *A. baumannii* infections. By using integrated computer-aided drug discovery pipelines, we have identified three promising anti-microbial inhibitors against *A. baumannii* as an initial step in novel drug development. All these inhibitors are the based on the scaffold of well-known drug Triclosan, which is an inhibitor of FabI protein in Gram-negative pathogens. Inhibitor 21272541 exhibiting the highest binding affinity with more compactness and stability against the *A. baumannii* FabI protein. This is similar to our recently report on *Klebsiella pneumonaie* which also identified inhibitor 21272541 as the most promising drug candidate against the FabI protein. Further development of inhibitor 21272541 is warranted to determine *in-vitro* and *in-vivo* activity against MDR Gram-negative organisms to overcome the greatest threat to human health.

### Ethical approval

The study is purely *in-silico*; hence no ethical clearance was required.

### Supplementary Information

Supplementary Figure 1.

Supplementary Figure 2.

Supplementary Table 3.

Supplementary Table 4.

Supplementary Table 5.

Supplementary Table 6.

Supplementary Table 7.

## Data Availability

The corresponding author can provide the data generated or used in this report on a reasonable requests.
