# Supplementary figures and images for: Structure-based identification of novel inhibitors targeting the enoyl-ACP reductase enzyme of Acinetobacter baumannii
Source: Sci Rep. 2023 Dec 4;13:21331. doi: 10.1038/s41598-023-48696-z (PMC10694131; doi:10.1038/s41598-023-48696-z)

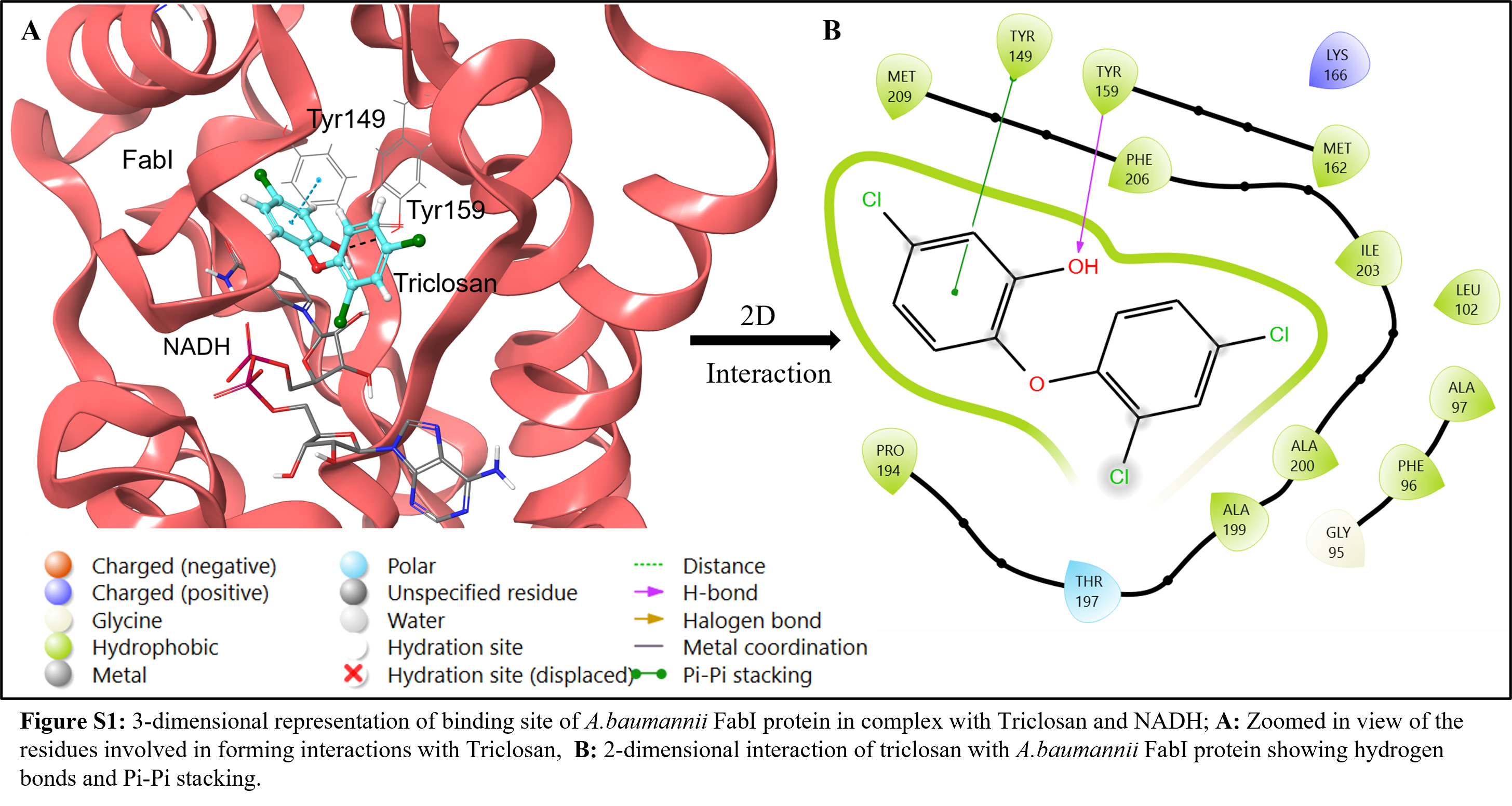

Supplement: Supplementary file 1 — Supplementary Figure 1. [file 41598_2023_48696_MOESM1_ESM.tif]

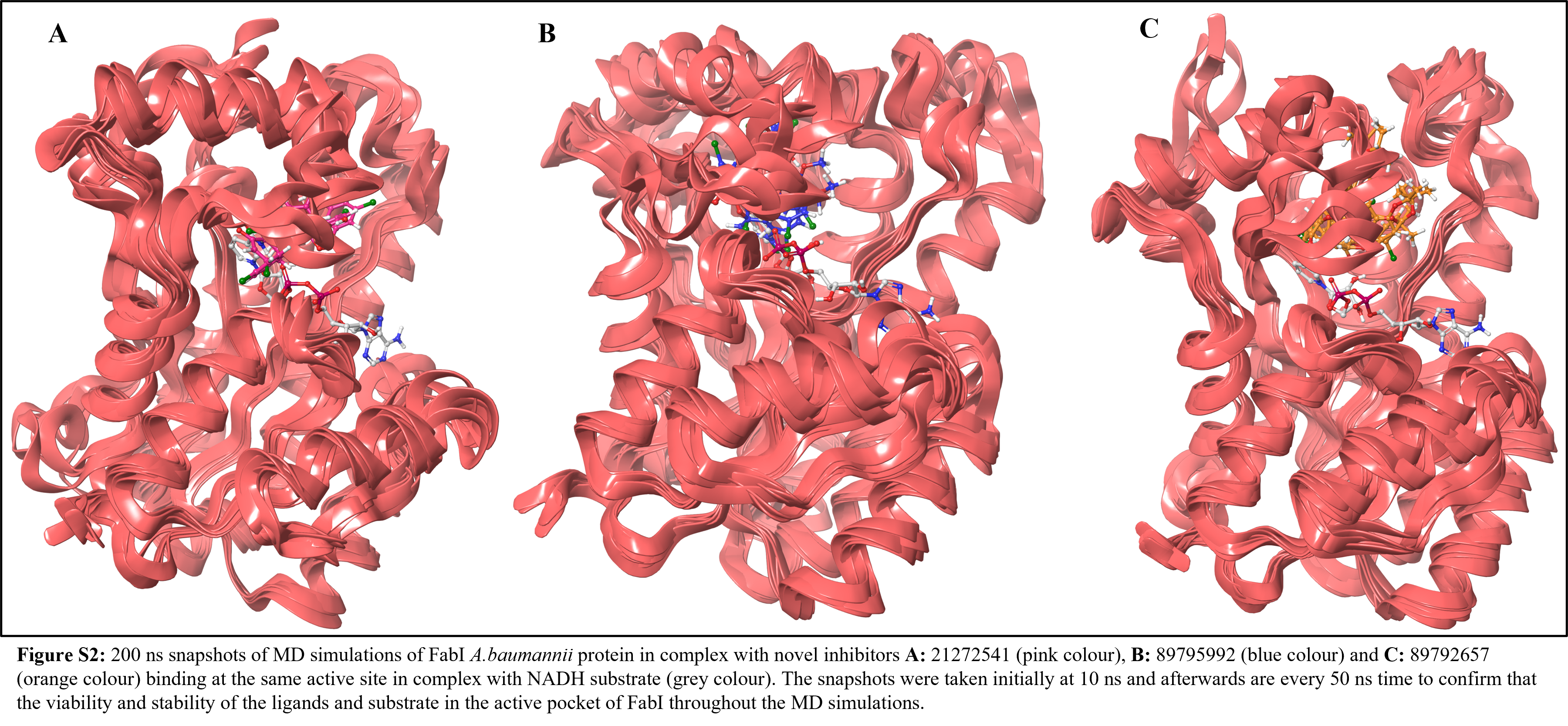

Supplement: Supplementary file 2 — Supplementary Figure 2. [file 41598_2023_48696_MOESM2_ESM.tif]
