# Supplementary material for: Structure-based identification of novel inhibitors targeting the enoyl-ACP reductase enzyme of Acinetobacter baumannii
Source: Sci Rep. 2023 Dec 4;13:21331. doi: 10.1038/s41598-023-48696-z (PMC10694131; doi:10.1038/s41598-023-48696-z)
Supplement: Supplementary file 3 — Supplementary Table 3. [file 41598_2023_48696_MOESM3_ESM.docx]

**Table S1:** PubChem IDs and smile structures of 140 compounds retrieved from the PubChem database.

| **S No. SMILES** |  | **PubChem IDs** |
| --- | --- | --- |
| 1. COc1cc(Cl)ccc1Oc1ccc(Cl)cc1O 2. [2H]c1c([2H])c(Oc2ccc(Cl)cc2[O-])c(Cl)c([2H])c1Cl 3. [O-]c1cc(Cl)ccc1Oc1ccc(Cl)cc1Cl 4. [O-]c1cc(Cl)ccc1O[13c]1[13cH][13cH][13c](Cl)[13cH][13c]1Cl 5. Oc1ccccc1Oc1c(O)cc(Cl)c(Cl)c1Cl 6. [O-]c1cc(Cl)cc(O)c1Oc1ccc(Cl)cc1Cl 7. C1=CC=C(C(=C1)O)OC2=C(C=CC(=C2[O])Cl)Cl 8. C1=CC=C(C(=C1)[O])OC2=C(C=CC(=C2O)Cl)Cl 9. Oc1ccccc1Oc1ccc(F)cc1Cl 10. Oc1cc(Cl)ccc1Oc1ccc(Cl)cc1 11. Oc1cc(Cl)ccc1OC1=CC[C@@H](Cl)C=C1Cl 12. Oc1cc(Cl)ccc1Oc1ccc(Cl)cc1O 13. Cc1ccc(Oc2c([O-])cc(Cl)cc2Cl)cc1 14. Oc1cc(Oc2ccc(Cl)cc2Cl)c(O)cc1Cl 15. Oc1ccccc1Oc1c(O)cccc1Cl 16. [O-]c1cc(Cl)ccc1Oc1ccc(Cl)cc1Cl 17. [O-]c1cc(Cl)ccc1Oc1ccc(Cl)cc1Cl 18. [O-]c1cc(Cl)ccc1Oc1ccc(Cl)cc1Cl 19. [O-]c1cc(Cl)ccc1Oc1ccc(Cl)cc1Cl 20. [O-]c1cc(Cl)ccc1Oc1ccc(Cl)cc1Cl 21. [O-]c1cc(Cl)ccc1Oc1ccc(Cl)cc1Cl 22. [O-]c1cc(Cl)ccc1Oc1ccc(Cl)cc1Cl 23. [O-]c1cc(Cl)ccc1Oc1ccc(Cl)cc1Cl 24. [O-]c1cc(Cl)ccc1Oc1ccc(Cl)cc1Cl 25. [O-]c1cc(Cl)ccc1Oc1ccc(Cl)cc1Cl 26. [O-]c1cc(Cl)ccc1Oc1ccc(Cl)cc1Cl 27. [O-]c1cc(Cl)ccc1Oc1ccc(Cl)cc1Cl 28. [O-]c1cc(Cl)ccc1Oc1ccc(Cl)cc1Cl 29. [O-]c1cc(Cl)ccc1Oc1ccc(Cl)cc1Cl 30. [O-]c1cc(Cl)ccc1Oc1ccc(Cl)cc1Cl 31. [O-]c1cc(Cl)ccc1Oc1ccc(Cl)cc1Cl 32. [O-]c1cc(Cl)ccc1Oc1ccc(Cl)cc1Cl 33. [O-]c1cc(Cl)ccc1Oc1ccc(Cl)cc1Cl 34. [O-]c1cc(Cl)ccc1Oc1ccc(Cl)cc1Cl 35. [O-]c1cc(Cl)ccc1Oc1ccc(Cl)cc1Cl 36. [O-]c1cc(Cl)ccc1Oc1ccc(Cl)cc1Cl 37. [O-]c1cc(Cl)ccc1Oc1ccc(Cl)cc1Cl 38. [O-]c1cc(Cl)ccc1Oc1ccc(Cl)cc1Cl 39. [O-]c1cc(Cl)ccc1Oc1ccc(Cl)cc1Cl 40. [O-]c1cc(Cl)ccc1Oc1ccc(Cl)cc1Cl 41. [O-]c1cc(Cl)ccc1Oc1ccc(Cl)cc1Cl 42. [O-]c1cc(Cl)ccc1Oc1ccc(Cl)cc1Cl 43. [O-]c1cc(Cl)ccc1Oc1ccc(Cl)cc1Cl 44. [O-]c1cc(Cl)ccc1Oc1ccc(Cl)cc1Cl 45. [O-]c1cc(Cl)ccc1Oc1ccc(Cl)cc1Cl 46. [O-][13c]1[13cH][13c](Cl)[13cH][13cH][13c]1O[13c]1[13cH][13cH][13c](Cl)[13cH][13c]1Cl 47. COc1ccccc1Oc1cc(Cl)ccc1O 48. [O-]c1c(Oc2ccc(Cl)c(Cl)c2O)ccc(Cl)c1Cl 49. [O-]c1c(Cl)cccc1Oc1cccc(Cl)c1Cl 50. [O-]c1c(Oc2ccc(Cl)cc2O)ccc(Cl)c1Cl 51. Oc1cc(Cl)ccc1Oc1ccccc1Cl 52. Oc1cc(Cl)ccc1Oc1ccccc1Cl 53. [O-]c1c(Cl)cc(Cl)cc1Oc1ccc(Cl)cc1Cl 54. Oc1ccccc1Oc1ccc(Cl)cc1Cl 55. Oc1cc(Cl)ccc1OC1=CC=CCC1(Cl)Cl 56. Oc1ccc(Oc2ccc(Cl)cc2O)c(Cl)c1 57. O=S(=O)([O-])Oc1cc(Cl)ccc1Oc1ccc(Cl)cc1Cl 58. Oc1ccccc1Oc1ccc(Cl)cc1 59. Oc1ccc(Cl)cc1Oc1cc(Cl)ccc1O 60. Oc1ccc(Cl)cc1Oc1ccc(Cl)cc1 61. Oc1cc(Cl)ccc1Oc1ccccc1 62. Oc1cc(Cl)ccc1Oc1ccccc1 63. [O-]c1cc(Cl)c(Cl)cc1Oc1ccc(Cl)cc1Cl 64. [O-]c1c(Oc2cc(Cl)c(Cl)cc2O)ccc(Cl)c1Cl 65. Oc1ccc(Cl)cc1Oc1cccc(Cl)c1 66. Oc1cccc(Cl)c1Oc1cccc(Cl)c1Cl 67. Oc1cc(Cl)cc(Cl)c1Oc1ccc(Cl)cc1 68. Oc1ccc(Cl)cc1Oc1cc(Cl)ccc1Cl 69. Oc1cc(Cl)ccc1Oc1cc(Cl)cc(Cl)c1 70. [O-]c1cc(Cl)c(I)cc1Oc1ccc(Cl)cc1Cl 71. Oc1cc(Cl)cc(Cl)c1Oc1ccccc1 72. Oc1cc(Cl)ccc1Oc1cc(Cl)ccc1Cl 73. [O-]c1c(Oc2ccc(Cl)cc2Cl)ccc(Cl)c1I 74. Oc1c(Cl)cccc1Oc1ccccc1 75. Oc1ccc(Oc2ccc(Cl)cc2Cl)cc1Cl 76. [O-]c1c(Cl)cccc1Oc1ccccc1Cl 77. [O-]c1c(Oc2ccccc2Cl)ccc(Cl)c1Cl 78. COc1ccc(Oc2ccc(Cl)cc2O)c(Cl)c1 79. [O-]c1c(Oc2ccc(Cl)cc2Cl)ccc(Cl)c1Cl 80. O=C([O-])Oc1cc(Cl)ccc1Oc1ccc(Cl)cc1Cl 81. CC(C)Oc1ccc(Oc2ccc(Cl)cc2O)c(Cl)c1 82. Oc1cc(Cl)ccc1Oc1ccc(Cl)c(Cl)c1 83. [O-]c1c(Cl)cccc1Oc1ccc(Cl)cc1Cl 84. [O-]c1ccc(Cl)c(Cl)c1Oc1ccc(Cl)cc1Cl 85. [O-]c1ccc(Cl)c(Cl)c1Oc1cccc(Cl)c1Cl 86. Clc1cc(Cl)c2c(c1)Oc1ccccc1O2 87. Clc1cc([37Cl])c2c(c1)Oc1ccccc1O2 88. Cl[13c]1[13cH][13c](Cl)[13c]2[13c]([13cH]1)O[13c]1[13cH][13cH][13cH][13cH][13c]1O2 89. Oc1c(Cl)cccc1OC1CCCCC1 90. Oc1ccc(Cl)c(Cl)c1Oc1ccccc1 91. Oc1cccc(Cl)c1Oc1ccccc1 92. Oc1cccc(Cl)c1OC1CCCCC1 93. Oc1cccc(Cl)c1Oc1ccc(Cl)cc1 94. Oc1cc(Cl)ccc1Oc1c(Cl)cccc1Cl 95. [O-]c1c(Oc2ccccc2)cc(Cl)c(Cl)c1Cl 96. [O-]c1cc(Cl)cc(Cl)c1Oc1ccc(Cl)cc1Cl 97. Oc1ccccc1Oc1cc(Cl)c(Cl)cc1Cl 98. Oc1cccc(Cl)c1Oc1ccccc1Cl 99. Oc1ccccc1Oc1cc(Cl)cc(Cl)c1 100. C1=CC(=C(C=C1Cl)Cl)OC2=C(C(=CC(=C2)Cl)Cl)[O] 101. Oc1ccc(Cl)cc1Oc1ccccc1 102. Oc1cccc(Cl)c1Oc1cc(Cl)ccc1Cl 103. Oc1cc(Cl)ccc1Oc1cccc(Cl)c1Cl 104. [O-]c1ccc(Cl)cc1Oc1cc(Cl)c(Cl)cc1Cl 105. Oc1c(Oc2ccccc2)ccc(Cl)c1Cl 106. Oc1cc(Cl)c(I)cc1Oc1ccc(Cl)cc1 107. Oc1ccccc1Oc1cc(Cl)c(Cl)c(Cl)c1Cl 108. Oc1c(Cl)cccc1Oc1cccc(Cl)c1O 109. CCOc1cc(Cl)ccc1Oc1ccc(Cl)cc1O 110. Oc1cc(Cl)c(Cl)c(Cl)c1Oc1ccccc1 111. Oc1ccccc1Oc1cccc(Cl)c1 112. COc1c(O)cc(Cl)cc1Cl 113. Oc1cc(Cl)c(Cl)cc1Oc1ccc(Cl)cc1 114. [O-]c1ccc(Cl)cc1Oc1ccc(Cl)cc1Cl 115. Oc1ccccc1Oc1cccc(Cl)c1Cl 116. [O-]c1c(Cl)c(Cl)c(Cl)c(Cl)c1Oc1ccccc1 117. CCCOc1c(O)cc(Cl)cc1Cl 118. Oc1ccccc1Oc1ccc(Cl)c(Cl)c1Cl 119. Oc1ccccc1Oc1ccc(Cl)c(Cl)c1Cl 120. Oc1ccc(Cl)cc1Oc1cc(Cl)cc(Cl)c1 121. COc1ccc(Cl)cc1Oc1cc(Cl)ccc1O 122. Oc1ccc(Oc2ccccc2O)cc1Cl 123. C1=CC(=C(C(=C1Cl)O)OC2=C(C=CC(=C2[O])Cl)Cl)Cl 124. COc1cc(Cl)cc(Cl)c1O 125. [O-]c1c(Cl)cc(Cl)cc1Oc1cc(Cl)cc(Cl)c1[O-] 126. CCOc1cc(Cl)cc(Cl)c1O 127. CC(C)Oc1c(O)cc(Cl)cc1Cl 128. [O-]c1cccc(Cl)c1Oc1ccc(Cl)cc1Cl 129. Oc1ccccc1Oc1c(Cl)c(Cl)c(Cl)c(Cl)c1Cl 130. Oc1ccccc1Oc1cc(Cl)cc(Cl)c1Cl 131. COc1cc(Cl)ccc1Oc1ccc(O)cc1Cl 132. Oc1ccccc1Oc1ccccc1Cl 133. [O-]c1c(Cl)cccc1Oc1ccc(O)cc1Cl 134. [O-]c1c(Cl)cccc1Oc1cccc(Cl)c1 135. [O-]c1cc(Cl)ccc1Oc1cc(Cl)c(Cl)cc1Cl 136. C=CCOc1c(O)ccc(Cl)c1Cl 137. CCOc1c(O)cc(Cl)cc1Cl 138. C=C(C)[C@@H]1CC=C(C)CC1 139. CCOc1c(O)cc(Cl)c(Cl)c1Cl 140. [O-]c1c(Oc2cc(Cl)c(Cl)c(Cl)c2[O-])cc(Cl)c(Cl)c1ClC1=CC(=C(C=C1Cl)O)OC2=C(C=C(C=C2)Cl)Cl | 89795992  45040608  25271835  76973291  87255639  67724551  102580654  102580655  141014847  18807  89126271  21272541  91264846  85840590  91122080  5564  138396115  161958069  161752747  161350492  161156022  160116095  157391163  144318390  144318384  122506975  88358443  87201530  70628635  70265497  70255327  69978356  69963777  69729527  68829664  68552661  68107534  67346643  67258653  66685457  66601504  18413505  18362548  22340835  67724550  101429827  56985515  157264116  70475583  21272522  162102454  17994679  3015664  11528970  60173044  21272512  67606152  12940631  71581338  12774296  5271320  91295715  165111  187307  11207551  68420804  20574907  18913824  13529054  162345191  15764571  12386541  162345199  69130964  20645734  101247244  70475976  89796023  173961  88175907  89792657  13529052  15897842  129853550  71335367  39727  14392135  101103377  72228339  18381077  22345063  68372481  12774298  23364922  21941275  20645735  21099545  20029653  22761252  101252616  12774295  134588942  18694998  71446197  20309156  85957727  139969477  19753595  163986661  6452320  19808418  92353  20574908  147298  14345695  176550  82267104  21975971  67743856  10401922  131976749  101466906  102580657  28051  154230063  119003677  82267072  15483970  156913  68418405  86084988  13266143  101466907  15861460  20309152  141727260  12905468  67858178  20267427  9822749 | -7.6345  -7.61938  -7.61938  -7.61938  -7.45871  -7.45459  -7.32747  -7.32747  -7.25159  -7.21989  -7.20442  -7.17164  -7.15987  -7.15581  -7.15153  -7.14041  -7.14041  -7.14041  -7.14041  -7.14041  -7.14041  -7.14041  -7.14041  -7.14041  -7.14041  -7.14041  -7.14041  -7.14041  -7.14041  -7.14041  -7.14041  -7.14041  -7.14041  -7.14041  -7.14041  -7.14041  -7.14041  -7.14041  -7.14041  -7.14041  -7.14041  -7.14041  -7.14041  -7.14041  -7.14041  -7.14041  -7.10356  -7.09735  -7.05519  -6.98214  -6.9383  -6.9383  -6.92726  -6.89277  -6.86  -6.85771  -6.80718  -6.79581  -6.76971  -6.76396  -6.75651  -6.75651  -6.73562  -6.73491  -6.66081  -6.6482  -6.55924  -6.52272  -6.49057  -6.48909  -6.47112  -6.45403  -6.44048  -6.42012  -6.40909  -6.40585  -6.3964  -6.39093  -6.36371  -6.35182  -6.31139  -6.296  -6.28134  -6.2693  -6.25946  -6.24995  -6.24995  -6.24995  -6.24846  -6.21064  -6.18871  -6.1504  -6.14652  -6.14162  -6.10043  -6.07111  -6.06374  -6.06092  -6.04027  -5.98379  -5.93293  -5.91579  -5.90438  -5.9035  -5.88569  -5.87065  -5.859  -5.85674  -5.85247  -5.80207  -5.79188  -5.7821  -5.7476  -5.74019  -5.72211  -5.69244  -5.64737  -5.64444  -5.64444  -5.62068  -5.6197  -5.55819  -5.54912  -5.53355  -5.50745  -5.48896  -5.47225  -5.46655  -5.42761  -5.40784  -5.40634  -5.39242  -5.38895  -5.32686  -5.27662  -5.27628  -5.17067  -5.14127  -4.84628  -1.0000 |
