# Supplementary material for: Structure-based identification of novel inhibitors targeting the enoyl-ACP reductase enzyme of Acinetobacter baumannii
Source: Sci Rep. 2023 Dec 4;13:21331. doi: 10.1038/s41598-023-48696-z (PMC10694131; doi:10.1038/s41598-023-48696-z)
Supplement: Supplementary file 5 — Supplementary Table 5. [file 41598_2023_48696_MOESM5_ESM.docx]

**Table S3:** High throughout virtual screening by Glide standard precision (SP) protocol resulted in 130 compounds.

| S. No. SMILES | PubChem ID | Docking score (kcal/mol) |
| --- | --- | --- |
| 1. COc1cc(Cl)ccc1Oc1ccc(Cl)cc1O 2. CC(C)Oc1ccc(Oc2ccc(Cl)cc2O)c(Cl)c1 3. Oc1cc(Cl)ccc1Oc1ccc(Cl)cc1O 4. COc1ccc(Oc2ccc(Cl)cc2[O-])c(Cl)c1 5. Oc1ccc(Oc2ccc(Cl)cc2O)c(Cl)c1 6. O[13c]1[13cH][13c](Cl)[13cH][13cH][13c]1O[13c]1[13cH][13cH][13c](Cl)[13cH][13c]1Cl 7. Oc1cc(Cl)ccc1Oc1ccc(Cl)cc1Cl 8. [2H]c1c([2H])c(Oc2ccc(Cl)cc2O)c(Cl)c([2H])c1Cl 9. [2H]Oc1cc(Cl)ccc1Oc1ccc(Cl)cc1Cl 10. Oc1cc(Cl)ccc1Oc1ccc(Cl)cc1Cl 11. Oc1cc(Cl)ccc1Oc1ccc(Cl)cc1Cl 12. Oc1cc(Cl)ccc1Oc1ccc(Cl)cc1Cl 13. Oc1cc(Cl)ccc1Oc1ccc(Cl)cc1Cl 14. Oc1cc(Cl)ccc1Oc1ccc(Cl)cc1Cl 15. Oc1cc(Cl)ccc1Oc1ccc(Cl)cc1Cl 16. Oc1cc(Cl)ccc1Oc1ccc(Cl)cc1Cl 17. Oc1cc(Cl)ccc1Oc1ccc(Cl)cc1Cl 18. Oc1cc(Cl)ccc1Oc1ccc(Cl)cc1Cl 19. Oc1cc(Cl)ccc1Oc1ccc(Cl)cc1Cl 20. Oc1cc(Cl)ccc1Oc1ccc(Cl)cc1Cl 21. Oc1cc(Cl)ccc1Oc1ccc(Cl)cc1Cl 22. Oc1cc(Cl)ccc1Oc1ccc(Cl)cc1Cl 23. Oc1cc(Cl)ccc1Oc1ccc(Cl)cc1Cl 24. Oc1cc(Cl)ccc1Oc1ccc(Cl)cc1Cl 25. Oc1cc(Cl)ccc1Oc1ccc(Cl)cc1Cl 26. Oc1cc(Cl)ccc1Oc1ccc(Cl)cc1Cl 27. Oc1cc(Cl)ccc1Oc1ccc(Cl)cc1Cl 28. Oc1cc(Cl)ccc1Oc1ccc(Cl)cc1Cl 29. Oc1cc(Cl)ccc1Oc1ccc(Cl)cc1Cl 30. Oc1cc(Cl)ccc1Oc1ccc(Cl)cc1Cl 31. Oc1cc(Cl)ccc1Oc1ccc(Cl)cc1Cl 32. Oc1cc(Cl)ccc1Oc1ccc(Cl)cc1Cl 33. Oc1cc(Cl)ccc1Oc1ccc(Cl)cc1Cl 34. Oc1cc(Cl)ccc1Oc1ccc(Cl)cc1Cl 35. Oc1cc(Cl)ccc1Oc1ccc(Cl)cc1Cl 36. Oc1cc(Cl)ccc1Oc1ccc(Cl)cc1Cl 37. Oc1cc(Cl)ccc1Oc1ccc(Cl)cc1Cl 38. Oc1cc(Cl)ccc1Oc1ccc(Cl)cc1Cl 39. Oc1cc(Cl)ccc1O[13c]1[13cH][13cH][13c](Cl)[13cH][13c]1Cl 40. Oc1cc(Cl)ccc1Oc1c(Cl)cccc1Cl 41. Oc1cc(Oc2ccc(Cl)cc2Cl)c(O)cc1Cl 42. [O-]c1cc(Cl)cc(Cl)c1Oc1ccc(Cl)cc1Cl 43. [O-]c1cc(Cl)ccc1Oc1ccc(Cl)c(Cl)c1O 44. Oc1cc(Cl)cc(O)c1Oc1ccc(Cl)cc1Cl 45. [O-]c1cc(Cl)c(Cl)c(Cl)c1Oc1ccccc1O 46. Oc1cc(Cl)ccc1Oc1ccccc1Cl 47. Oc1cc(Cl)ccc1Oc1ccccc1Cl 48. [O-]c1cc(Cl)ccc1OC1=CC=CCC1(Cl)Cl 49. Oc1cc(Cl)ccc1Oc1cc(Cl)c(Cl)cc1Cl 50. Oc1cc(Cl)ccc1OC1=CC[C@@H](Cl)C=C1Cl 51. [O-]c1ccccc1Oc1ccc(F)cc1Cl 52. Oc1cc(Cl)c(Cl)cc1Oc1ccc(Cl)c(Cl)c1O 53. [O-]c1c(Oc2ccc(Cl)c(Cl)c2O)ccc(Cl)c1Cl 54. [O-]c1cc(Cl)ccc1Oc1cccc(Cl)c1Cl 55. [O-]c1cc(Cl)ccc1Oc1cc(Cl)cc(Cl)c1 56. Oc1cccc(Cl)c1Oc1ccc(Cl)cc1Cl 57. [O-]c1cc(Cl)ccc1Oc1ccc(Cl)cc1 58. COc1cc(Cl)ccc1Oc1ccc(O)cc1Cl 59. [O-]c1cccc(Cl)c1Oc1ccccc1O 60. [O-]c1ccccc1Oc1cc(Cl)cc(Cl)c1Cl 61. [O-]c1ccccc1Oc1ccc(Cl)cc1Cl 62. Oc1cc(Cl)ccc1Oc1ccc(Cl)c(Cl)c1 63. [O-]c1cc(Cl)c(Cl)cc1Oc1ccc(Cl)cc1Cl 64. COc1ccccc1Oc1cc(Cl)ccc1O 65. Oc1cc(Cl)ccc1Oc1ccccc1 66. Oc1cc(Cl)ccc1Oc1ccccc1 67. Cc1ccc(Oc2c([O-])cc(Cl)cc2Cl)cc1 68. Oc1c(Cl)cccc1Oc1cccc(Cl)c1O 69. Oc1cc(Cl)ccc1Oc1cc(Cl)ccc1Cl 70. [O-]c1ccccc1Oc1ccc(Cl)c(Cl)c1Cl 71. [O-]c1ccccc1Oc1ccc(Cl)c(Cl)c1Cl 72. [O-]c1cc(Cl)c(Cl)cc1Oc1ccc(Cl)cc1 73. Oc1ccc(Cl)cc1Oc1cc(Cl)ccc1O 74. Oc1ccc(Oc2cccc(Cl)c2O)c(Cl)c1 75. [O-]c1ccc(Cl)cc1Oc1ccc(Cl)cc1Cl 76. [O-]c1c(Oc2ccc(Cl)cc2Cl)ccc(Cl)c1Cl 77. CCOc1cc(Cl)ccc1Oc1ccc(Cl)cc1[O-] 78. Oc1ccccc1Oc1ccccc1Cl 79. Oc1ccccc1Oc1cc(Cl)c(Cl)cc1Cl 80. [O-]c1c(Cl)cc(Cl)cc1Oc1ccc(Cl)cc1Cl 81. [O-]c1cc(Cl)cc(Cl)c1Oc1ccc(Cl)cc1 82. [O-]c1ccccc1Oc1cccc(Cl)c1Cl 83. Oc1c(Cl)cccc1Oc1ccc(Cl)cc1Cl 84. Oc1c(Cl)cccc1Oc1cccc(Cl)c1Cl 85. Oc1ccccc1Oc1ccc(Cl)cc1 86. [O-]c1cc(Cl)cc(Cl)c1Oc1ccccc1 87. [O-]c1ccccc1Oc1cc(Cl)cc(Cl)c1 88. Oc1ccc(Cl)c(Cl)c1Oc1ccccc1 89. [O-]c1cc(Cl)c(I)cc1Oc1ccc(Cl)cc1Cl 90. [O-]c1ccc(Cl)c(Cl)c1Oc1ccc(Cl)cc1Cl 91. [O-]c1c(Cl)cccc1Oc1ccccc1Cl 92. [O-]c1ccc(Cl)cc1Oc1cccc(Cl)c1 93. [O-]c1ccc(Cl)cc1Oc1ccc(Cl)cc1 94. [O-]c1cccc(Cl)c1Oc1cccc(Cl)c1Cl 95. Oc1ccccc1Oc1c(Cl)c(Cl)c(Cl)c(Cl)c1Cl 96. O=S(=O)([O-])Oc1cc(Cl)ccc1Oc1ccc(Cl)cc1Cl 97. Oc1c(Oc2ccccc2Cl)ccc(Cl)c1Cl 98. Oc1ccc(Oc2ccccc2O)cc1Cl 99. [O-]c1c(Oc2ccccc2)ccc(Cl)c1Cl 100. [O-]c1cc(Cl)c(Cl)c(Cl)c1Oc1ccccc1 101. Oc1c(Cl)cccc1Oc1cccc(Cl)c1 102. Oc1c(Oc2cc(Cl)c(Cl)c(Cl)c2O)cc(Cl)c(Cl)c1Cl 103. [O-]c1cccc(Cl)c1Oc1ccccc1Cl 104. [O-]c1cccc(Cl)c1Oc1ccccc1 105. [O-]c1c(Oc2ccc(Cl)cc2Cl)ccc(Cl)c1I 106. [O-]c1cc(Cl)c(I)cc1Oc1ccc(Cl)cc1 107. [O-]c1ccc(Cl)cc1Oc1cc(Cl)ccc1Cl 108. [O-]c1c(Cl)cccc1Oc1ccccc1 109. Oc1ccccc1Oc1cccc(Cl)c1 110. COc1ccc(Cl)cc1Oc1cc(Cl)ccc1O 111. [O-]c1ccc(Cl)cc1Oc1cc(Cl)cc(Cl)c1 112. [O-]c1cccc(Cl)c1Oc1cc(Cl)ccc1Cl 113. CC(C)Oc1c([O-])cc(Cl)cc1Cl 114. Oc1ccc(Cl)cc1Oc1cc(Cl)c(Cl)cc1Cl 115. Oc1ccc(Cl)cc1Oc1ccccc1 116. Oc1ccc(Cl)c(Cl)c1Oc1cccc(Cl)c1Cl 117. [O-]c1ccccc1Oc1cc(Cl)c(Cl)c(Cl)c1Cl 118. Clc1cc(Cl)c2c(c1)Oc1ccccc1O2 119. Clc1cc([37Cl])c2c(c1)Oc1ccccc1O2 120. Cl[13c]1[13cH][13c](Cl)[13c]2[13c]([13cH]1)O[13c]1[13cH][13cH][13cH][13cH][13c]1O2 121. Oc1c(Cl)c(Cl)c(Cl)c(Cl)c1Oc1ccccc1 122. [O-]c1ccc(Oc2ccc(Cl)cc2Cl)cc1Cl 123. Oc1c(Oc2ccccc2)cc(Cl)c(Cl)c1Cl 124. O=C(O)Oc1cc(Cl)ccc1Oc1ccc(Cl)cc1Cl 125. [O-]c1c(Cl)cccc1OC1CCCCC1 126. Oc1cccc(Cl)c1OC1CCCCC1 127. CCOc1c([O-])cc(Cl)cc1Cl 128. CCCOc1c([O-])cc(Cl)cc1Cl 129. COc1cc(Cl)cc(Cl)c1[O-] 130. [O-]c1cccc(Cl)c1Oc1ccc(Cl)cc1 | 89795992  89792657  21272541  89796023  21272512  101429827  5564  45040608  138396115  161958069  161752747  161350492  161156022  160116095  157391163  144318390  144318384  122506975  88358443  87201530  70628635  70265497  70255327  69978356  69963777  69729527  68829664  68552661  68107534  67346643  67258653  66685457  66601504  25271835  18413505  18362548  22340835  67724550  76973291  23364922  85840590  20645735  21272522  67724551  87255639  162102454  17994679  60173044  20309152  89126271  141014847  187307  157264116  18694998  13529054  15483970  18807  86084988  91122080  68418405  11528970  13529052  165111  56985515  5271320  91295715  91264846  19753595  12386541  21975971  67743856  20574908  71581338  101466907  147298  173961  163986661  13266143  21099545  3015664  20574907  14345695  15897842  70475583  12940631  15764571  22761252  18381077  162345191  129853550  101247244  11207551  12774296  68420804  156913  67606152  70475976  101466906  20309156  6452320  15861460  9822749  20029653  22345063  162345199  85957727  18913824  69130964  19808418  131976749  10401922  134588942  82267072  71446197  12774295  71335367  139969477  39727  14392135  101103377  176550  20645734  21941275  88175907  72228339  68372481  12905468  82267104  28051  12774298 | -8.31638  -8.29075  -8.08632  -7.9758  -7.93955  -7.90991  -7.90035  -7.90035  -7.90035  -7.90035  -7.90035  -7.90035  -7.90035  -7.90035  -7.90035  -7.90035  -7.90035  -7.90035  -7.90035  -7.90035  -7.90035  -7.90035  -7.90035  -7.90035  -7.90035  -7.90035  -7.90035  -7.90035  -7.90035  -7.90035  -7.90035  -7.90035  -7.90035  -7.90035  -7.90035  -7.90035  -7.90035  -7.90035  -7.90035  -7.8958  -7.8924  -7.80107  -7.79415  -7.73043  -7.72792  -7.71428  -7.71428  -7.64864  -7.60764  -7.60582  -7.57411  -7.57018  -7.55717  -7.5315  -7.51346  -7.49658  -7.48506  -7.47536  -7.44727  -7.34789  -7.32796  -7.31066  -7.30608  -7.30139  -7.2845  -7.2845  -7.2787  -7.26579  -7.25993  -7.23387  -7.23387  -7.21289  -7.20673  -7.20135  -7.19466  -7.18603  -7.18537  -7.17165  -7.16794  -7.13754  -7.12825  -7.10665  -7.08534  -7.04069  -7.02064  -7.00465  -7.00312  -6.98336  -6.97861  -6.97123  -6.97009  -6.96206  -6.95904  -6.93105  -6.86892  -6.86175  -6.803  -6.78376  -6.77024  -6.7625  -6.75562  -6.74391  -6.73856  -6.72601  -6.69862  -6.67614  -6.65496  -6.63226  -6.57651  -6.55864  -6.52722  -6.47589  -6.43506  -6.43396  -6.41692  -6.35649  -6.34554  -6.34284  -6.33495  -6.33495  -6.30058  -6.3  -6.29619  -6.29615  -6.22673  -6.22567  -6.16504  -6.12384  -6.11996  -6.02425 |
