# Supplementary material for: Structure-based identification of novel inhibitors targeting the enoyl-ACP reductase enzyme of Acinetobacter baumannii
Source: Sci Rep. 2023 Dec 4;13:21331. doi: 10.1038/s41598-023-48696-z (PMC10694131; doi:10.1038/s41598-023-48696-z)
Supplement: Supplementary file 6 — Supplementary Table 6. [file 41598_2023_48696_MOESM6_ESM.docx]

**Table S4:** High throughout virtual screening protocol of Glide XP.

| S No. SMILES | PubChem ID | Docking score (kcal/mol) |
| --- | --- | --- |
| 1. Oc1cc(Cl)ccc1Oc1ccc(Cl)cc1O 2. COc1cc(Cl)ccc1Oc1ccc(Cl)cc1O 3. CC(C)Oc1ccc(Oc2ccc(Cl)cc2O)c(Cl)c1 4. Oc1ccccc1Oc1c(O)cc(Cl)c(Cl)c1Cl 5. COc1ccc(Oc2ccc(Cl)cc2O)c(Cl)c1 6. Oc1cc(Cl)ccc1OC1=CC[C@@H](Cl)C=C1Cl 7. Oc1cc(Oc2ccc(Cl)cc2Cl)c(O)cc1Cl 8. Oc1cc(Cl)ccc1Oc1cccc(Cl)c1Cl 9. Oc1cc(Cl)ccc1Oc1ccccc1Cl 10. Oc1cc(Cl)ccc1Oc1ccccc1Cl 11. Oc1cc(Cl)ccc1OC1=CC=CCC1(Cl)Cl 12. Oc1cc(Cl)ccc1Oc1ccc(Cl)c(Cl)c1 13. Oc1cc(Cl)ccc1Oc1c(Cl)cccc1Cl 14. Oc1ccc(Oc2ccc(Cl)cc2O)c(Cl)c1 15. Oc1ccc(Cl)cc1Oc1cc(Cl)ccc1O 16. Oc1cc(Cl)ccc1Oc1cc(Cl)cc(Cl)c1 17. Oc1cc(Cl)ccc1Oc1ccc(Cl)cc1 18. Oc1ccccc1Oc1ccc(F)cc1Cl 19. Oc1cc(Cl)ccc1Oc1ccccc1 20. Oc1cc(Cl)ccc1Oc1ccccc1 21. Oc1ccccc1Oc1cc(Cl)c(Cl)cc1Cl 22. Oc1ccccc1Oc1ccc(Cl)cc1Cl 23. Oc1ccccc1Oc1c(O)cccc1Cl 24. Oc1ccccc1Oc1ccccc1Cl 25. [O-]c1cc(Cl)cc(O)c1Oc1ccc(Cl)cc1Cl 26. [O-]c1cc(Cl)ccc1Oc1ccc(Cl)cc1Cl 27. [O-]c1cc(Cl)ccc1O[13c]1[13cH][13cH][13c](Cl)[13cH][13c]1Cl 28. [O-]c1cc(Cl)ccc1Oc1ccc(Cl)cc1Cl 29. [O-]c1cc(Cl)ccc1Oc1ccc(Cl)cc1Cl 30. [O-]c1cc(Cl)ccc1Oc1ccc(Cl)cc1Cl 31. [O-]c1cc(Cl)ccc1Oc1ccc(Cl)cc1Cl 32. [O-]c1cc(Cl)ccc1Oc1ccc(Cl)cc1Cl 33. [O-]c1cc(Cl)ccc1Oc1ccc(Cl)cc1Cl 34. [O-]c1cc(Cl)ccc1Oc1ccc(Cl)cc1Cl 35. [O-]c1cc(Cl)ccc1Oc1ccc(Cl)cc1Cl 36. [O-]c1cc(Cl)ccc1Oc1ccc(Cl)cc1Cl 37. [O-]c1cc(Cl)ccc1Oc1ccc(Cl)cc1Cl 38. [O-]c1cc(Cl)ccc1Oc1ccc(Cl)cc1Cl 39. [O-]c1cc(Cl)ccc1Oc1ccc(Cl)cc1Cl 40. [O-]c1cc(Cl)ccc1Oc1ccc(Cl)cc1Cl 41. [O-]c1cc(Cl)ccc1Oc1ccc(Cl)cc1Cl 42. [O-]c1cc(Cl)ccc1Oc1ccc(Cl)cc1Cl 43. [O-]c1cc(Cl)ccc1Oc1ccc(Cl)cc1Cl 44. [O-]c1cc(Cl)ccc1Oc1ccc(Cl)cc1Cl 45. [O-]c1cc(Cl)ccc1Oc1ccc(Cl)cc1Cl 46. [O-]c1cc(Cl)ccc1Oc1ccc(Cl)cc1Cl 47. CCO.C1=CC(=C(C=C1Cl)O)OC2=C(C=C(C=C2)Cl)Cl 48. [13CH]1=[13CH][13C](=[13C]([13CH]=[13C]1Cl)O)O[13C]2=[13C]([13CH]=[13C]([13CH]=[13CH]2)Cl)Cl 49. COc1cc(Cl)ccc1Oc1ccc(O)cc1Cl 50. [O-]c1cc(Cl)ccc1Oc1cc(Cl)c(Cl)cc1Cl 51. [O-]c1cc(Cl)ccc1Oc1ccc(Cl)cc1Cl 52. [2H]c1c([2H])c(Oc2ccc(Cl)cc2[O-])c(Cl)c([2H])c1Cl 53. [O-]c1cc(Cl)ccc1Oc1ccc(Cl)cc1Cl 54. [O-]c1cc(Cl)ccc1Oc1ccc(Cl)cc1Cl 55. [O-]c1cc(Cl)ccc1Oc1ccc(Cl)cc1Cl 56. [O-]c1cc(Cl)ccc1Oc1ccc(Cl)cc1Cl 57. [O-]c1cc(Cl)ccc1Oc1ccc(Cl)cc1Cl 58. [O-]c1cc(Cl)ccc1Oc1ccc(Cl)cc1Cl 59. [O-]c1cc(Cl)ccc1Oc1ccc(Cl)cc1Cl 60. [O-]c1cc(Cl)ccc1Oc1ccc(Cl)cc1Cl 61. [O-]c1cc(Cl)ccc1Oc1ccc(Cl)cc1Cl 62. [O-]c1cc(Cl)ccc1Oc1ccc(Cl)cc1Cl 63. [O-]c1cc(Cl)cc(Cl)c1Oc1ccc(Cl)cc1Cl 64. [O-]c1c(Oc2ccc(Cl)cc2Cl)ccc(Cl)c1Cl 65. [O-]c1cccc(Cl)c1Oc1ccc(Cl)cc1Cl 66. [O-]c1c(Oc2ccc(Cl)c(Cl)c2O)ccc(Cl)c1Cl 67. COc1ccccc1Oc1cc(Cl)ccc1O 68. [O-]c1c(Oc2ccc(Cl)cc2O)ccc(Cl)c1Cl 69. [O-]c1c(Oc2cc(Cl)c(Cl)cc2O)ccc(Cl)c1Cl 70. [O-]c1c(Cl)cc(Cl)cc1Oc1ccc(Cl)cc1Cl 71. [O]c1cc(Cl)c(Cl)cc1Oc1ccc(Cl)cc1ClOc1ccc(Oc2ccc(Cl)cc2O)c(Cl)c1 | 21272541  89795992  89792657  87255639  89796023  89126271  85840590  18694998  162102454  17994679  60173044  13529052  23364922  21272512  71581338  13529054  18807  141014847  5271320  91295715  21099545  11528970  91122080  13266143  67724551  25271835  76973291  88358443  87201530  70628635  70265497  70255327  69978356  69963777  69729527  68829664  68552661  68107534  67346643  67258653  66685457  66601504  18413505  18362548  22340835  67724550  101429827  86084988  20309152  5564  45040608  138396115  161958069  161752747  161350492  161156022  160116095  157391163  144318390  144318384  122506975  20645735  173961  15483970  157264116  56985515  21272522  187307  3015664  165111 | -9.84609  -9.6564  -9.56968  -9.08894  -8.99319  -8.87509  -8.81453  -8.76418  -8.76051  -8.76051  -8.70228  -8.63565  -8.57473  -8.54755  -8.53518  -8.52954  -8.36764  -8.28555  -8.23255  -8.23255  -8.17167  -8.13369  -8.03359  -7.79486  -7.61587  -7.48249  -7.48249  -7.41559  -7.41559  -7.41559  -7.41559  -7.41559  -7.41559  -7.41559  -7.41559  -7.41559  -7.41559  -7.41559  -7.41559  -7.41559  -7.41559  -7.41559  -7.41559  -7.41559  -7.41559  -7.41559  -7.41559  -7.38222  -7.26715  -7.18684  -7.18684  -7.18684  -7.18684  -7.18684  -7.18684  -7.18684  -7.18684  -7.18684  -7.18684  -7.18684  -7.18684  -7.0753  -7.02816  -6.86211  -6.82406  -6.20167  -5.82912  -5.52614  -4.12144  -3.98595 |
