# Supplementary material for: Structure-based identification of novel inhibitors targeting the enoyl-ACP reductase enzyme of Acinetobacter baumannii
Source: Sci Rep. 2023 Dec 4;13:21331. doi: 10.1038/s41598-023-48696-z (PMC10694131; doi:10.1038/s41598-023-48696-z)
Supplement: Supplementary file 7 — Supplementary Table 7. [file 41598_2023_48696_MOESM7_ESM.docx]

**Table S5:** XP protocol generated one pose for each ligand with total 23 compounds.

| S No. SMILES | PubChem ID | XP Score (kcal/mol) |
| --- | --- | --- |
| 1. Oc1cc(Cl)ccc1Oc1ccc(Cl)cc1O 2. COc1cc(Cl)ccc1Oc1ccc(Cl)cc1O 3. CC(C)Oc1ccc(Oc2ccc(Cl)cc2O)c(Cl)c1 4. Oc1ccccc1Oc1c(O)cc(Cl)c(Cl)c1Cl 5. COc1ccc(Oc2ccc(Cl)cc2O)c(Cl)c1 6. Oc1cc(Cl)ccc1OC1=CC[C@@H](Cl)C=C1Cl 7. Oc1cc(Oc2ccc(Cl)cc2Cl)c(O)cc1Cl 8. Oc1cc(Cl)ccc1Oc1cccc(Cl)c1Cl 9. Oc1cc(Cl)ccc1Oc1ccccc1Cl 10. Oc1cc(Cl)ccc1Oc1ccccc1Cl 11. Oc1cc(Cl)ccc1OC1=CC=CCC1(Cl)Cl 12. Oc1cc(Cl)ccc1Oc1ccc(Cl)c(Cl)c1 13. Oc1cc(Cl)ccc1Oc1c(Cl)cccc1Cl 14. Oc1ccc(Oc2ccc(Cl)cc2O)c(Cl)c1 15. Oc1ccc(Cl)cc1Oc1cc(Cl)ccc1O 16. Oc1cc(Cl)ccc1Oc1cc(Cl)cc(Cl)c1 17. Oc1cc(Cl)ccc1Oc1ccc(Cl)cc1 18. Oc1ccccc1Oc1ccc(F)cc1Cl 19. Oc1cc(Cl)ccc1Oc1ccccc1 20. Oc1cc(Cl)ccc1Oc1ccccc1 21. Oc1ccccc1Oc1cc(Cl)c(Cl)cc1Cl 22. Oc1ccccc1Oc1ccc(Cl)cc1Cl 23. Oc1ccccc1Oc1c(O)cccc1Cl | 21272541  89795992  89792657  87255639  89796023  89126271  85840590  18694998  162102454  17994679  60173044  13529052  23364922  21272512  71581338  13529054  18807  141014847  5271320  91295715  21099545  11528970  91122080 | -9.84609  -9.6564  -9.56968  -8.99294  -8.99319  -8.87509  -8.81453  -8.76418  -8.76051  -8.76051  -8.70228  -8.63565  -8.57473  -8.54755  -8.53518  -8.52954  -8.36764  -8.28555  -8.23255  -8.23255  -8.17167  -8.13369  -8.03359 |
